# Evaluating the impact of a multi-year produce prescription program on fruit and vegetable intake, and food security in south central New York

**DOI:** 10.1186/s12966-026-01955-4

**Published:** 2026-07-28

**Authors:** Therese Martin Cheteu Wabo, Kate Miller-Corcoran, Erin Summerlee, Genevive R. Meredith

**Affiliations:** 1 Department of Public and Ecosystem Health, Cornell University, Ithaca, NY USA; 2 Rural Health Network of South Central New York, Binghamton, NY USA; 3 Cornell Health Impacts Core, NY Ithaca, United States

**Keywords:** Produce Prescription Program, Food Security, Fruit and vegetable intake, Health

## Abstract

**Background:**

Rural communities experience high rates of diet-related chronic diseases, driven by limited access to healthy food, poor diet quality, and high food insecurity. Produce Prescription (PRx) programs aim to improve fruit and vegetable access and intake, alleviate food insecurity, yet evidence of their sustained impacts remains limited.

**Objective:**

To evaluate the short-and long-term impacts of a PRx program in rural New York State on fruit and vegetable intake, food security, self-reported health, and diet quality, with complementary insights into participants' experiences and program engagement.

**Methods:**

This multi-year evaluation included 1400 low-income participants with diet-related chronic diseases recruited from 2022 to 2024. The matched samples ranged from 117 to 261 participants per year, with a longitudinal cohort of 187 participants. Over three seasons, participants received farm shares or vouchers ($15/week for ~ 24 weeks) to purchase fruits and vegetables. Surveys assessed fruit and vegetable intake (times/day), food security, self-rated diet quality, and health status. Program use and engagement were measured through redemption data and patient feedback. The Wilcoxon and Stuart-Maxwell tests were used for year-by-year comparisons, multivariable mixed-effects models for longitudinal changes across three seasons, and thematic analysis of participants’ feedback.

**Results:**

The yearly analysis revealed small-to-moderate increases in fruit and vegetable intake and a moderate reduction in the mean number of affirmative responses, along with a significant shift towards higher food security categories. The mixed-effects models showed a sustained within-person increases in fruit, fruit juice, green vegetables, and other vegetables, with mean intake frequency rising by 0.27 (95% CI, 0.07 to 0.46), 0.33 (95% CI, 0.03 to 0.64), 0.17 (95% CI, 0.06 to 0.27), and 0.26 (95% CI, 0.11 to 0.40) times/day, respectively. The cumulative reduction in affirmative responses was -0.75 (95% CI, -1.11 to -0.39), and the odds of being in a more secure food category more than doubled across all intervention points compared to baseline, peaking at the 2024 pre-intervention timepoint, AOR = 3.06 (95% CI, 1.76 to 5.34). A redistribution toward marginal and high food security was evident, though low food security remained persistent. Improvements in self-rated health and diet quality were modest and inconsistent. Program engagement was high, with over 93% of participants receiving vouchers, and 65% redeemed them more than 10 times/season. Participants appreciated how PRx improved access to and affordability of fresh produce and suggested increasing the number of vendors, the voucher amounts, and extending the program duration.

**Conclusion:**

This evaluation showed both short- and long-term improvements in fruit and vegetable intake and food security, with greater benefits observed with continued participation. However, improvements in food security reflected reduced severity rather than a full transition to food security, underscoring the need for supplementary structural supports.

**Supplementary Information:**

The online version contains supplementary material available at https://doi.org/10.1186/s12966-026-01955-4.

## Background

Cardiometabolic diseases, including diabetes and obesity, contribute to premature deaths and high healthcare costs in the United States (U.S.) [1, 2]. These conditions are largely preventable and are strongly influenced by dietary behaviors and intake [3]. Poor diet quality is one of the leading modifiable risk factors for chronic diseases such as diabetes, obesity, cardiovascular diseases, and hypertension [4]. In the U.S., diet-related chronic diseases disproportionally affect low-income populations, especially in rural areas, where geographic and economic barriers restrict access to healthy foods [5, 6], weakening the prevention and management of chronic diseases [7]. Household food insecurity (FI)—a household-level economic and social condition in which limited financial resources constrain consistent access to adequate and nutritious food—has been strongly linked with poor diet quality, increased healthcare utilization, and adverse health outcomes [8]. In 2023, 15.4% of U.S. rural households reported being food insecure (vs. 13.5% of all U.S households) [9, 10].

To improve food access and diet quality in the U.S., many programs exist. These include benefits or entitlement programs that increase access to an array of foods (e.g., the Supplemental Nutrition Assistance Program (SNAP), the Special Supplemental Nutrition Program for Women and Children (WIC), free school breakfast or lunch programs), food access and education programs (e.g., the Expanded Food and Nutrition Education Program (EFNEP)), and community-based services (e.g., food pantries, food donation programs, etc.). While this array of services aims to help alleviate food insecurity, promote healthier eating behaviors, and reduce nutrition-related health disparities [11], evidence suggests these programs often fall short in improving diet quality or addressing the root causes of nutrition-related chronic diseases [12, 13]; this may be because they operate in isolation from healthcare systems, provide limited support for purchasing fresh foods, and/or are not adapted to community- or population-specific needs [14].

In recent years, healthcare systems and policymakers have begun to recognize the role of “Food as Medicine,” emphasizing the benefits of integrating nutrient-rich food access initiatives into clinical care [14]. The Produce Prescription (PRx) Program is one food as medicine model whereby healthcare providers screen and refer eligible patients to a social service provider that helps the patient increase access to and use of fruits and vegetables in their diet by addressing cost and access barriers [14]. Depending on the PRx model, the ‘prescription’ may entail outreach and education, subsidized access to fruits and vegetables through vouchers that are redeemable at local grocery stores, farm stands, or farmers' markets, and education and behavior-change support [15]. PRx programs aim to increase access to produce, promote healthy dietary behaviors, improve food security (FS), and diet-related health outcomes [16].

Most PRx program evaluations have reported improvements in fruits and vegetable intake and reductions in food insecurity. In a systematic review of 20 PRx studies conducted between 2012 and 2023, participants generally demonstrated improved dietary behaviors, and Harper et al. also concluded that PRx programs provide an opportunity to improve food security among populations [17]. However, the existing evidence on PRx programs is largely drawn from interventions conducted over a single season, leaving limited evidence about the sustained impact across repeated program exposure. In addition, relatively few evaluations have explored behavioral factors related to food choices, shopping practices, and perceptions of diet quality [18–21]. To fill these gaps and inform the design of ongoing PRx programs, we evaluated the short- and long-term impacts of the rural health network's PRx program on fruit and vegetable Intake, food security, self-reported health status, and perceived diet quality. We further integrated complementary qualitative insights into participants’ experiences and behavioral factors shaping the program engagement. Our analytic framework combined year-by-year pre-post comparisons to capture immediate program impact and mixed-effects modeling to account for individual-level variation in longitudinal analyses and the program's long-term impact.

### New York State diet-related health needs

Cardiometabolic diseases are the leading cause of premature deaths in New York State (NYS) [22]. Public health priorities to address this need include improving Food Security and diet quality, particularly in rural NYS communities where geographic and economic barriers limit access to healthy foods [23]. For example, in NYS in 2022, some 69% of rural households experienced some form of food insecurity [24], and only 12.% of adults statewide reported consuming at least two or more fruits and three or more vegetables daily [25].

The southern tier of NYS, comprising Broome, Chemung, Chenango, Delaware, Schuyler, Steuben, Tioga, and Tompkins counties, is a predominantly rural region [26], including many that are designated as “transitional distressed area” based on high poverty, low income, and high unemployment rates [23]. In these communities, there is limited access to nutrient-rich foods, particularly for low-income residents living far from grocery stores. The food environment index, a measure of access to healthy foods and affordability, is at or below the NYS average, 8.6, across these counties, indicating significant barriers to food access [27]. Broome faces some of the most severe socioeconomic disparities among NYS counties, with 18.9% of the population living below the poverty, and 15.2% experiencing food insecurity [28, 29].

## Methods

### Population and setting

The study participants included 1,400 adults aged 18 years or older who participated in the PRx Program in NYS across Broome, Delaware, Tioga, and Tompkins counties (PRxSCNY) from 2022 to 2024. Patients were eligible to participate in the program if they were seen by a participating provider and (a) had or were at risk of a diet-related chronic disease and (b) were lower-income (e.g., eligible for Medicaid, WIC, SNAP) or screened for food insecurity. PRxSCNY uses a partnership model in which clinicians at partner agencies screen patients for eligibility and, with patient consent, refer them to PRxSCNY for program enrollment. Participants choose to receive a weekly Community Supported Agriculture (CSA) share (~ 24 weeks, mid-June to November), or receive $120 worth of vouchers at each of three clinic visits (up to $360 total for the season) [30]. Participants who opted for CSA/farm share received a weekly produce box containing a variety of seasonal produce (e.g., leafy greens, root vegetables, tomatoes, apples). Box contents varied by season and availability and were sourced from local farms. Additional details on program design and implementation are provided in Supplementary file 1.

### Data collection

Every participant who opted in via their provider’s visit completed a structured survey before and after the intervention. The surveys captured sociodemographic data (age, gender, race/ethnicity, county of residence, household size and composition, benefits use (e.g., SNAP), employment status, and type of health insurance), along with fruit and vegetable frequency intake, food security, perceived general health status, and diet quality using a consistent set of questions.

Post-surveys additionally included behavioral measures related to food choices, shopping behaviors, program engagement, and program perception assessed using closed- and open-ended questions. Surveys were administered in person, online, by phone, or on paper, depending on participant preference.

### Outcomes

- Changes in Fruit and Vegetable Intake (times/day, continuous). The Dietary Screener Questionnaire (DSQ) was used to assess the fruit and vegetable intake of all participants. They reported how often they consumed these foods per month, per week, or per day; responses were then converted to a daily frequency. Frequency measures capture changes in eating patterns, such as how often fruits and vegetables are incorporated into the diet, which is a relevant behavioral outcome. These measures do not capture portion size, total quantity, or energy-adjusted intake. In 2022, only five items (fruits, green vegetables, other vegetables, potatoes, and fried potatoes) were assessed. In 2023 and 2024, five other items were added to the survey (100% fruit juice, beans, salsa, pizza, and tomato sauce).
- Changes in Food Security. Food security was assessed using the 6-item Household Food Security Survey Module (HFSSM). Participants' responses were coded as affirmative or non-affirmative. The sum of affirmative responses to the six questions constituted the household’s raw food security score, with a higher number of affirmations reflecting greater food insecurity. For analyses, food security was used both as a continuous variable to capture incremental changes over time and as a categorical variable (high, marginal, low, very low) [31] to examine meaningful transitions in food security levels.
- Changes in Health and Diet Quality. Participants self-reported their overall health and diet quality on an ordinal 5-point Likert scale (Excellent, Very Good, Good, Fair, Poor). The questions were: (1) Would you say that, in general, your health is…? and (2) Would you say that, in general, your diet is…? These single-item measures have been widely used in population health and nutrition research and are considered strong, valid predictors of general health [32] and diet quality [33].
- Program perceptions and participant feedback: Participants responded to two open-ended questions: “What did you like best about the PRx Program?” and “What ideas do you have to make the PRx Program better?” Qualitative responses were analyzed using thematic content analysis to identify common themes of satisfaction, perceived benefits, and suggestions for improvement.

### Statistical analysis

#### Year-by-year analytic approach

Outcomes were measured at baseline and after program participation. For the year-by-year analysis, outcomes of interest included (a) changes in fruit and vegetable intake (all items); (b) changes in food security score and status; (c) changes in self-reported diet quality, and (d) changes in self-reported health status. We also explored behavioral measures and program perception.

Within-person changes of continuous variables were assessed using the Wilcoxon signed-rank test, and between-person changes for unpaired comparisons were examined using the Wilcoxon rank-sum test. Effect sizes for within and between-person comparisons were calculated as |r|= Z/√N, where Z is the test statistic and N the number of non-zero paired observations, and interpreted using conventional thresholds for small (≥ 0.1), moderate (≥ 0.3), and large (≥ 0.5) effects as previously described [34]. The Stuart-Maxwell test was used to evaluate within-person changes in self-reported health and diet quality. Qualitative feedback from open-ended survey responses was thematically coded and summarized by frequency.

#### Longitudinal analytic approach

##### Inclusion criteria

A participant was included in the analysis if they enrolled in two or more seasons and had at least four data-entry points (four program waves). For the longitudinal analysis, outcomes of interest included (a) changes in fruit and vegetable intake (four items) and (b) changes in food security and changes in self-rated diet quality and health.

We used a mixed-effects linear regression to analyze changes in fruit and vegetable intake frequency and food security (continuous), and a mixed-effects ordered logistic regression to analyze changes in food security (categorical) and self-rated diet and health. Both models included a random intercept for each participant to account for repeated measures over time. Intervention time was modeled as a categorical fixed effect, capturing differences across the six intervention points: 2022, 2023, and 2024 (pre- and post-intervention). Two models were estimated: an unadjusted model (model 1) and an adjusted model (model 2), which controlled for age, gender, ethnicity, employment status, county of residence, insurance status, household size, the presence of children under 17 years, and SNAP (recoded into ever received SNAP vs never received SNAP). This modeling approach allowed us to estimate time-specific changes in outcomes at each intervention time point while accounting for within-subject correlation. Marginal predicted values were plotted to visualize trends across the program years. All analyses were performed using Stata version 18.

## Results

### Participant characteristics by year of program enrollment (2022, 2023, 2024)

The PRx program enrolled 421, 526, and 453 participants in 2022, 2023, and 2024 across 18, 24, and 20 health care provider sites, respectively. Post-completion survey rates were 69.7%, 81.6%, and 76.6%, with matched pairs available for 117, 261, and 211 participants, respectively (Table 1). The mean age was 55.6(± 14.26), 54.4(± 13.82), and 56.0(± 14.72) years. Most participants were female (72–77.9%) and identified as white (71.5–74.6%) or black/African American (14.5–18.3%). Household sizes were predominantly small, with about 41–46% having 2–3 persons and 37.5–39.0% single-person households, and most had no children under 17 (73–77%). The majority were unable to work (35.0–43.7%) or retired (19.2–22.1%), and 58–63% received SNAP benefits. Matched samples tended to be older and differed modestly in racial composition across years.

**Table 1:** Participant characteristics(2022- 2024)

2022						2023					2024				
CHARACTERISTICS	Enrollment (*N* = 417)		Matched Pre-Post Data (*N* = 117)			Enrollment (*N* = 526)		Matched Pre-Post Data (*N* = 261)			Enrollment (*N* = 453)		Matched Pre-Post Data (*N* = 211)		
	N	Percent (%)	N	Percent (%)	% Retention	N	Percent (%)	N	Percent (%)	% Retention	N	Percent (%)	N	Percent (%)	% Retention
Age	Mean (SD): 55.62 (± 14.26)		Mean (SD): 59.08 (± 13.82**)**		28.10	Mean (SD): 54.40 (± 14.72)		Mean (SD): 56.19 (± 14.32)		49.62	Mean (SD): 55.97(± 15.42)		Mean (SD): 57.39(± 14.69)		46.58
GENDER															
Male	113	26.84	31	26.49	27.43	125	23.76	60	22.99	48.00	86	18.98	40	18.96	46.51
Female	303	71.97	85	72.64	28.05	389	73.95	196	75.10	50.39	353	77.92	166	78.67	47.03
ETHNICITY															
White	314	74.58	94	80.34	29.94	381	72.43	191	73.18	50.13	324	71.52	154	72.99	47.53
Black/African American	62	14.73	12	10.26	19.35	76	14.45	47	18.00	61.84	83	18.32	34	16.11	40.96
Hispanic white	21	4.98	6	5.13	28.57	41	7.79	13	4.98	31.71	22	4.6	11	5.21	50.00
Other ethnicity	24	5.70	5	4.27	20.83	88	16.73	5	1.91	5.68	24	5.30	7	3.32	29.17
HOUSEHOLD SIZE															
1	158	37.53	45	38.46	28.48	203	38.59	108	41.38	53.20	176	38.85	88	41.71	50.00
2–3	193	45.84	52	44.44	26.94	220	41.83	106	40.61	48.18	185	40.83	83	39.34	44.86
> = 4	70	16.63	20	17.09	28.57	103	19.58	47	18.01	45.63	92	20.31	40	18.96	43.48
CHILDREN < 17															
None	324	76.95	93	79.49	28.70	384	73.00	200	76.63	52.08	333	73.51	163	77.25	48.95
1–2	67	15.91	16	13.67	23.88	102	19.39	46	17.62	45.10	78	17.22	32	15.17	41.03
> = 3	33	7.83	8	6.84	24.24	40	7.60	15	5.75	37.50	42	9.27	16	7.58	38.10
SNAP BENEFITS															
Currently receiving SNAP	245	58.19	69	58.97	28.16	319	60.65	158	60.54	49.53	277	62.81	139	65.88	50.18
EMPLOYMENT															
Unable to work	184	43.71	54	46.15	29.35	184	34.98	95	36.40	51.63	152	33.55	65	30.81	42.76
Retired	92	21.85	34	29.06	36.96	101	19.20	54	20.69	53.47	100	22.08	57	27.01	57.00
Employed	85	20.19	16	13.67	18.82	132	25.09	62	23.75	45.93	113	24.94	45	21.33	39.82
Not employed	40	9.50	9	7.69	22.50	81	15.40	39	14.94	48.15	62	13.69	26	12.32	41.94
Disabled	13	3.08	2	1.71	15.38	11	2.09	4	1.53	44.44	9	1.99	5	2.37	55.56
Other	7	1.66	7	5.98	100	17	3.23	6	2.68	35.29	17	3.75	13	6.16	76.47

Matched Pre-Post Data: Data for participants who completed both pre and post-surveys

*Baseline Characteristics for three-year longitudinal Analysis (2022–2024):* Two baseline timepoints were included: 119 participants were enrolled in 2022, and 68 were newly enrolled in 2023, for a total of 187 unique participants. Each participant contributed multiple repeated measurements, resulting in a total of 858 observations. The mean age at baseline was 59.81 years (± 13.05) for 2022 enrollees and 54.81 years (± 14.79) for 2023 enrollees. Most were female (75.80%), White (75.40%), and 14.4% were Black/African American. At baseline, 42.8% of participants lived in 2–3 person households, 77.1% had no children under 17, 39.7% were currently receiving SNAP benefits, and 33.2% had previously received SNAP. Most respondents were unable to work (45.25%), and 27.35% were retired (Table 2).

**Table 2:** Baseline characteristics of participants included in the longitudinal cohort

Baseline characteristics	2022 (N = 119)	2023 (N = 68)	TOTAL (N/%)
AGE (mean ± SE)	59.81 ± 13.05	54.81 ± 14.79	-
GENDER			
Male	31	14	45(24.19)
Female	87	54	141(75.80)
ETHNICITY			
White	93	48	141(75.40)
Black/African American	13	14	27(14.4)
Hispanic white	6	3	9(4.8)
Other ethnicity	7	3	10(5.3)
HOUSEHOLD SIZE			
1	48	25	73(39)
2–3	52	28	80(42.8)
> = 4	19	15	34(18.2)
CHILDREN < 17			
None	97	48	145(77.1)
1–2	14	12	26(13.8)
> = 3	9	8	17(9)
SNAP BENEFITS			
Currently w/snap	73	0	73(39.7)
Never received snap	24	25	49(26.6)
Former snap	19	42	61(33.2)
Application pending	1	0	1(0.5)
EMPLOYMENT			
Unable to work	58	23	81(45.25)
Retired	33	16	49(27.35)
Employed	18	21	39(21.78)
Not employed	10	0	10(5.59)
Disabled	0	0	-
Other employment status	0	0	-
INSURANCE STATUS			
Yes	116	64	180(96.0)
No	0	3	3(1.60)
COUNTY			
Broome	71	40	111(59.35)
Delaware	29	13	42(22.4)
Tioga	11	6	17(9.09)
Tompkins	6	8	14(7.49)

### Outcomes

#### Changes in fruit and vegetable consumption

##### Year-by-year

***Fruit and vegetable consumption:*** The Independent pre-post comparison showed modest differences in fruit and vegetable consumption before and after the program participation. Participants consistently reported higher fruit and vegetable intake across program years, with significant differences observed in 2022 and 2023 for fruits, green vegetables, and other vegetables, and in 2024 for fruits and green vegetables. Effect sizes were very small (all *r* < 0.09, *p* < 0.05) (Table S1).

Year-by-year changes in the consumption of fruits, vegetables, and other food categories before and after the intervention among matched participants are summarized in Table 3. In 2022, fruit intake increased from a mean of 0.64(± 0.57) to 0.83(± 0.61) times/day (*p* < 0.01,* r* = *-*0.29), and green vegetables also increased from 0.33(± 0.36) to 0.42(± 0.41) (*p* < *0.01*, *r* = *-*0.27), both indicating small to moderate improvements. Intake of other vegetables rose from 0.57(± 0.48) to 0.66(± 0.50) but did not reach statistical significance (*p* = 0.06; *r* = 0.17). In 2024, fruit intake increased from 0.97 (± 1.15) to 1.02 (± 0.86) times/day (*p* = *0.01*; *r* = *-*0.17), corresponding to a small effect size, whereas no significant changes in vegetable intake were observed. No significant changes in fruit and vegetable Intake were observed in 2023.

**Table 3:** Year-specific comparison of daily frequency intake by food category before and after the program among matched participants

2022								2023						2024					
Food Category	Survey	Median	Mean (SD)	N	Z	Effect size	*P-value*	Median	Mean (SD)	N	*Z*	Effect Size	*P-value *	Median	Mean (SD)	N	*Z*	Effect Size	*P*-value
Fruits	Pre	0.50	0.64(±.057)	115	-3.11	-0.29	<0.01	0.50	1.06(±1.01)	253	0.16	0.01	0.87	0.50	0.97(±1.15)	205	-2.49	-0.17	0.01
	Post	0.78	0.83(±0.61)	115				0.78	0.92(±0.81)	253				0.78	1.02(±0.86)	205			
Green Vegetables	Pre	0.28	0.33(±0.36)	114	2.94	0 .27	<0.01	0.50	0.58(±9.70)	251	-0.13	0.01	0.89	0.39	0.53(±0.58)	200	-1.39		0.16
	Post	0.28	0.42(±0.41)	114				0.50	0.52(±0.53)	251				0.50	0.56(±0.58)	200			
Fried Potatoes	Pre	0.08	0.17(±0.21)	116	-3.03	-0.28	<0.01	0.08	0.16(±0.23)	252	-0.37	0.02	0.70	0.08	0.24(±0.44)	204	2.90	0.20	<0.001
	Post	0.14	0.24(±0.29)	116				0.08	0.17(±0.24)	252				0.08	0.20(±0.37)	204			
Potatoes	Pre	0.14	0.26(±0.35)	116	-1.46	-0.13	0.14	0.14	0.23(±0.24)	255	-0.21	0.01	0.83	0.14	0.26(±0.39)	203	0.26		0.79
	Post	0.21	0.28(±0.29)	116				0.14	0.23(±0.22)	255				0.14	0.25(±0.34)	203			
Other Vegetables	Pre	0.50	0.57(±0.48)	116	-1.88	-0.17	0.06	0.50	0.73(±0.70)	251	-0.93	0.06	0.35	0.50	0.73(±0.75)	206	-1.52		0.12
	Post	0.50	0.66(±0.50)	116				0.50	0.73(±0.60)	251				0.50	0.77(±0.65)	206			
Beans	Pre							0.08	0.23(±0.37)	194	1.20	0.09	0.22	0.08	0.20(±0.34)	162	-0.64		0.52
	Post							0.08	0.20(±0.29)	194				0.08	0.20(±0.33)	162			
Pizza	Pre							0.03	0.08(±0.11)	197	1.37	0.10	0.17	0.03	0.09(±0.22)	158	1.38		0.17
	Post							0.03	0.07(±0.10)	197				0.03	0.09(±0.29)	158			
Tomato sauce	Pre							0.08	0.18(±0.20)	169	-0.012	0.001	0.99	0.08	0.20(±0.32)	158	0.09		0.92
	Post							0.08	0.19(±0.26)	169				0.08	0.19(±0.31)	158			
Fruit Juice	Pre							0.08	1.15(±1.45)	230	-0.55	-0.04	0.58	0.43	1.21(±1.49)	186	0.32		0.75
	Post							0.08	1.81(±1.45)	230				0.08	1.24(±1.49)	186			
Salsa	Pre							0.03	0.08(±0.15)	212	1.04	0.07	0.29	0.03	0.08(±0.29)	171	0.27		0.79
	Post							0.00	0.09(±0.31)	212				0.00	0.08(±0.24)	171			

#### Changes in other food consumption

**N**o consistent differences were observed for other food categories between participants. Among matched participants, fried potato intake increased in 2022 from 0.17 (± 0.21) to 0.24 (± 0.29) times/day (*p* < 0.01, *r* = −0.28) with a borderline moderate effect size increase, whereas in 2024, intake decreased from 0.24(± 0.44) to 0.20(± 0.37) times/day (*p* < 0.001, *r* = 0.20), indicating a borderline moderate reduction. No changes in other food consumption were observed in 2023.

#### Changes in fruit and vegetable consumption over time

**Fig. 1:**
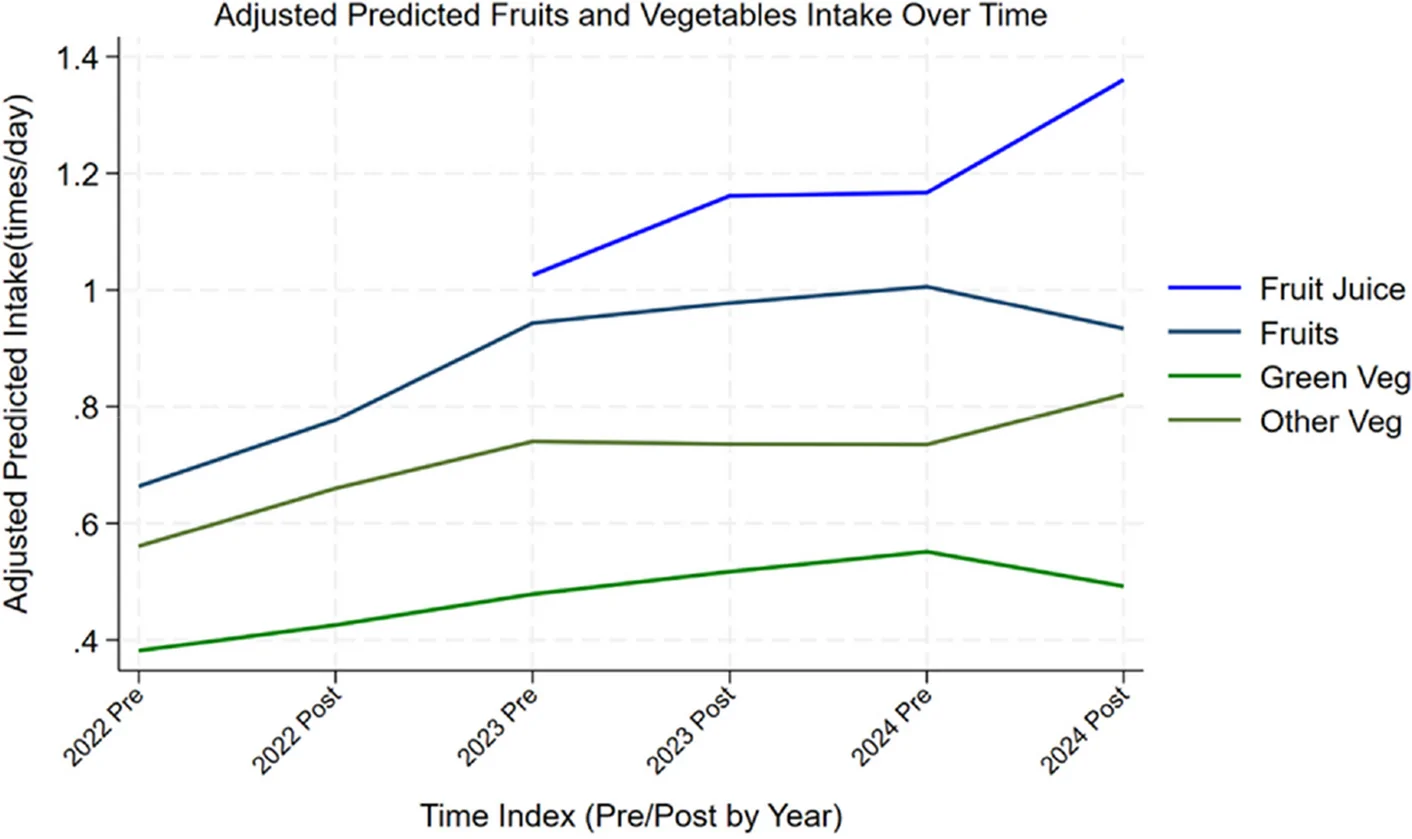
Longitudinal mixed effects model estimates of adjusted predicted fruits and Vegetables across three program seasons

In the mixed effects models, fruit and vegetable intake increased consistently over time (Table S2, Fig. 1). Overall, tests for linear trend indicated significant increases in fruits, green vegetables, other vegetables, and 100% fruit juice (adjusted p-trend < 0.05 for all).

The adjusted baseline mean intake of fruit was 0.66 times/day, which increased to 0.93 times/day by 2024 post-intervention, corresponding to a cumulative increase of 0.27 times/day over 3 seasons (95% CI 0.07 to 0.46).

For 100% fruit juice, adjusted frequency intake rose from 1.03(± 0.11) to 1.36(± 0.13), corresponding to a cumulative two-season increase of 0.33 times/day (95%CI 0.03 to 0.64).

Other vegetable intake also showed a consistent and significant increase, rising from 0.56 (± 0.05) to 0.82 (± 0.06), with a cumulative increase of 0.26 times/day (95% CI 0.11 to 0.40) across three seasons, representing the most consistent improvement.

Green vegetables increased from 0.38(± 0.04) to 0.49(± 0.05) times/day, peaking in 2024 pre-intervention 0.17 (95%CI 0.06 to 0.27) with attenuation at 2024 post-intervention (*p* = 0.06). Across the fruits and vegetables assessed, intraclass correlation coefficients (ICCs) ranged from 0.24 to 0.32, indicating that 68–76% of the variability was attributable to within-person changes over time (Table S4)**.**

### Other food consumption

No significant changes were observed over time**.**

#### *Changes in* food security

##### Year by year

**Fig. 2:**
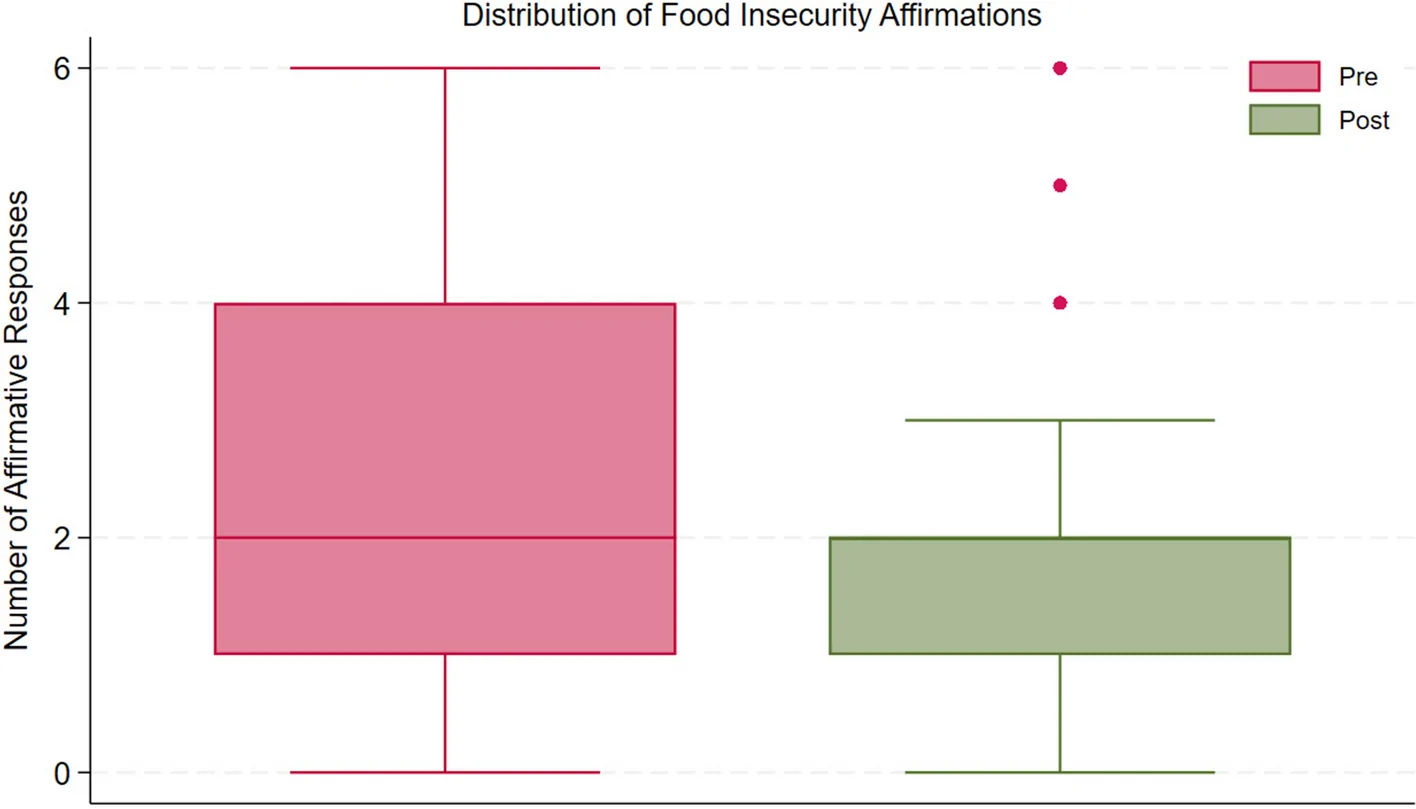
Food Security affirmative response Pre- and Post-program Among Matched Participants (2022)

**Fig. 3:**
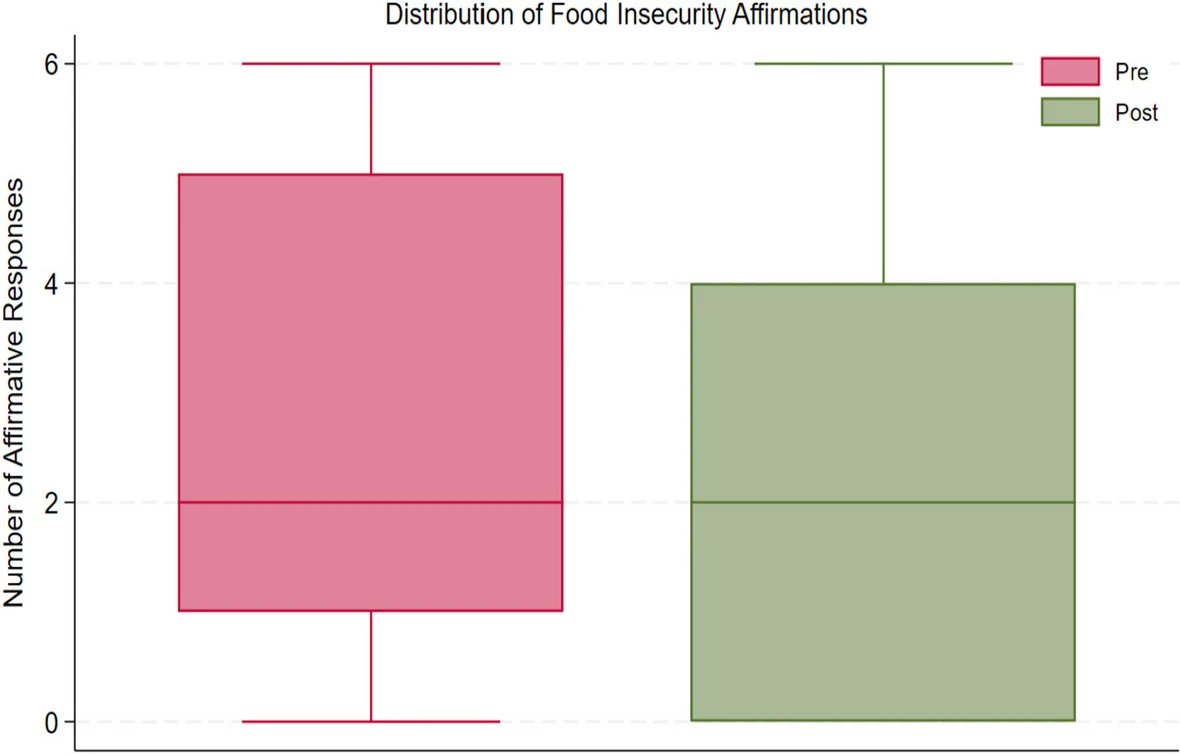
Food security affirmative responses Pre- and Post-program Among matched Participants (2023)

Household food security improved significantly among PRx program participants through 2023, then attenuated in 2024. Independent pre- and post-intervention comparisons indicated a reduction in affirmative responses to household food security items among participants. In 2022, 2023, and 2024, the mean responses were 2.24 ± 2.10 versus 2.89 ± 2.28, 1.90 ± 1.57 versus 2.33 ± 1.90, and 2.03 ± 1.62 versus 2.26 ± 1.85, respectively, with small effect sizes (*r* < 0.14). Year-by-year changes in the number of affirmative responses to household food security items among matched participants are shown in Figs. 2 and 3. In 2022, the mean number of affirmative responses decreased from 2.60 to 2.11 (*p* < 0.001; *r* = 40). In 2023, a significant difference was also observed, with the mean decreasing from 2.33 to 1.96 (*p* < 0.001; *r* = 0.40). This indicates moderate within-person improvements after intervention in 2022 and 2023, but not in 2024. Despite the within-person improvements observed after the intervention in 2022 and 2023, the mean remains within the low food security range (2–4 affirmations), even though values approached the marginal range in 2023 post-intervention (1.96 affirmations).

**Fig. 4:**
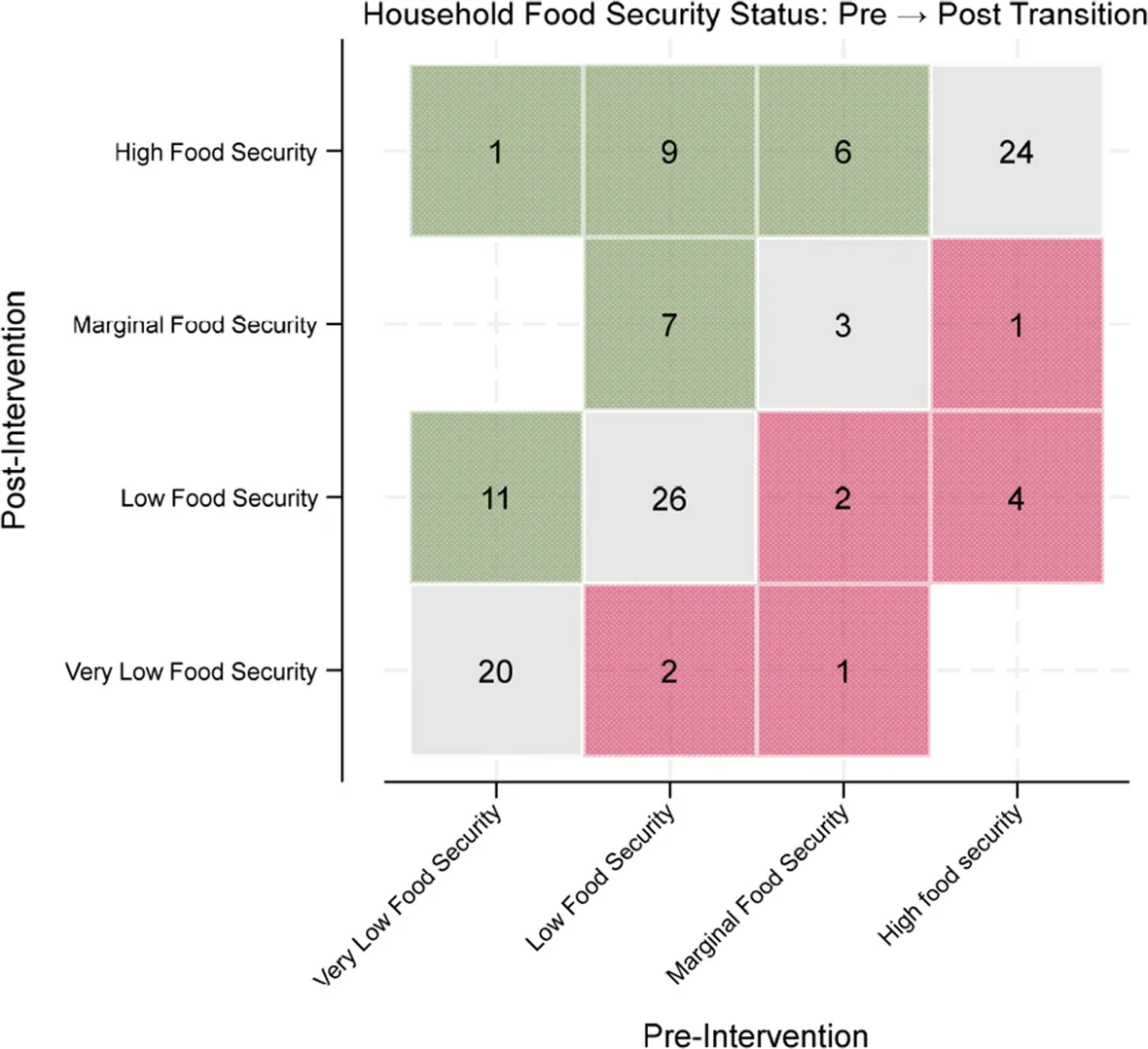
Food security status transitions among matched participants (2022)

**Fig. 5:**
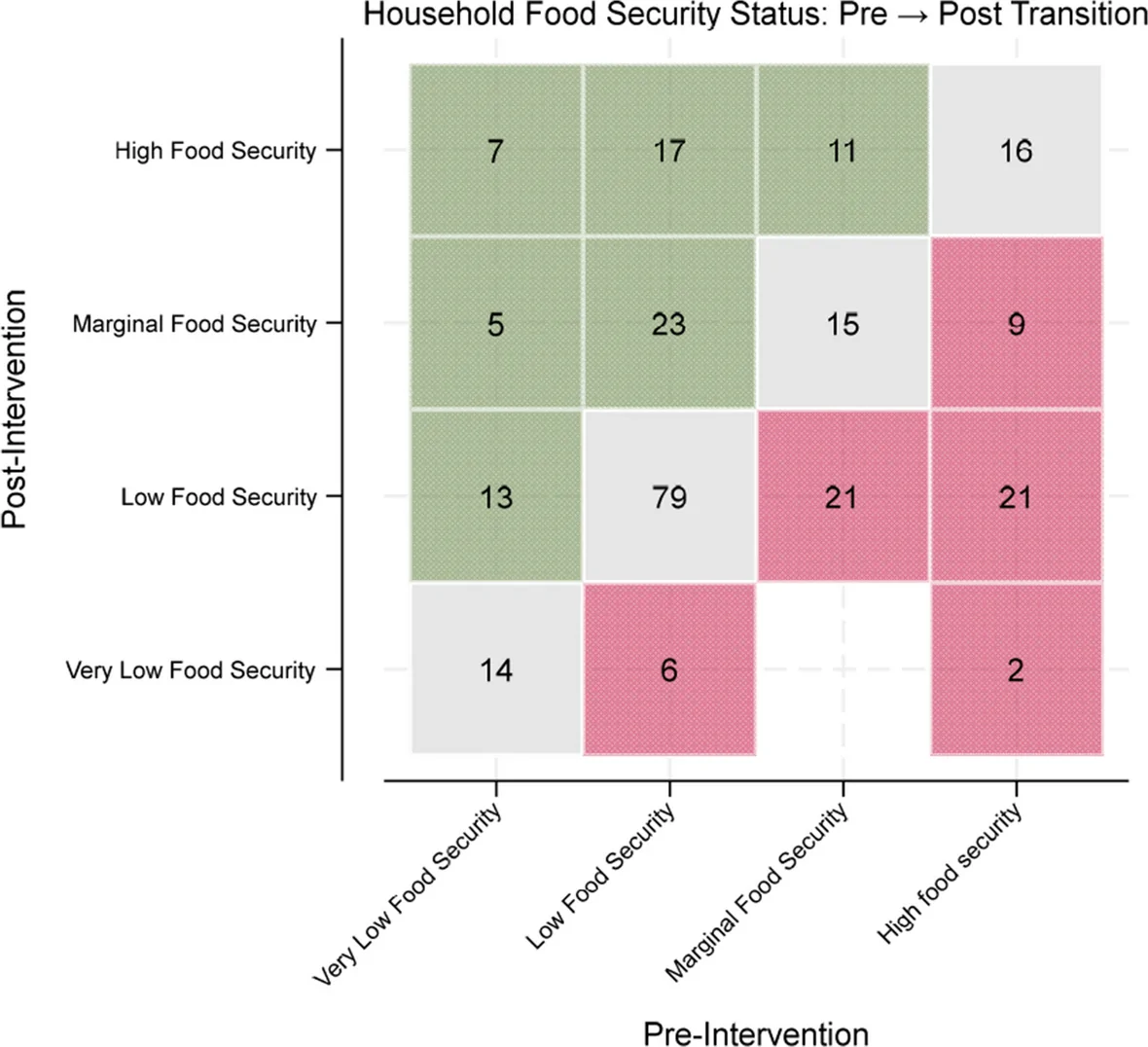
Food security status transitions among matched participants (2023)

Transition patterns across household food security categories showed both improvements and persistent food insecurity (Figs. 4 and 5). Among matched participants in 2022, transitions were.

predominantly towards higher food security categories, with 29.1% of participants improving their food security status, 8.5% experiencing a decline, and 62.4% remaining in the same category. Improvements were observed across all food insecurity categories.. In 2023, 29.34% of participants transitioned to improved food security, with upward shifts observed from marginal, low, and very low food security towards higher categories. Overall, food insecurity persisted among 80.3% of participants, of whom 23% experienced a worsening in food security status. Consistent with this transition pattern, the Stuart-Maxwell test indicated a significant shift in the distribution of food security status in 2022 and 2023 (*p* < 0.05). In contrast, no significant shifts were observed in 2024, where improvements and declines were more balanced across categories following the intervention (Table S3).

#### Changes in food security over time

**Fig. 6:**
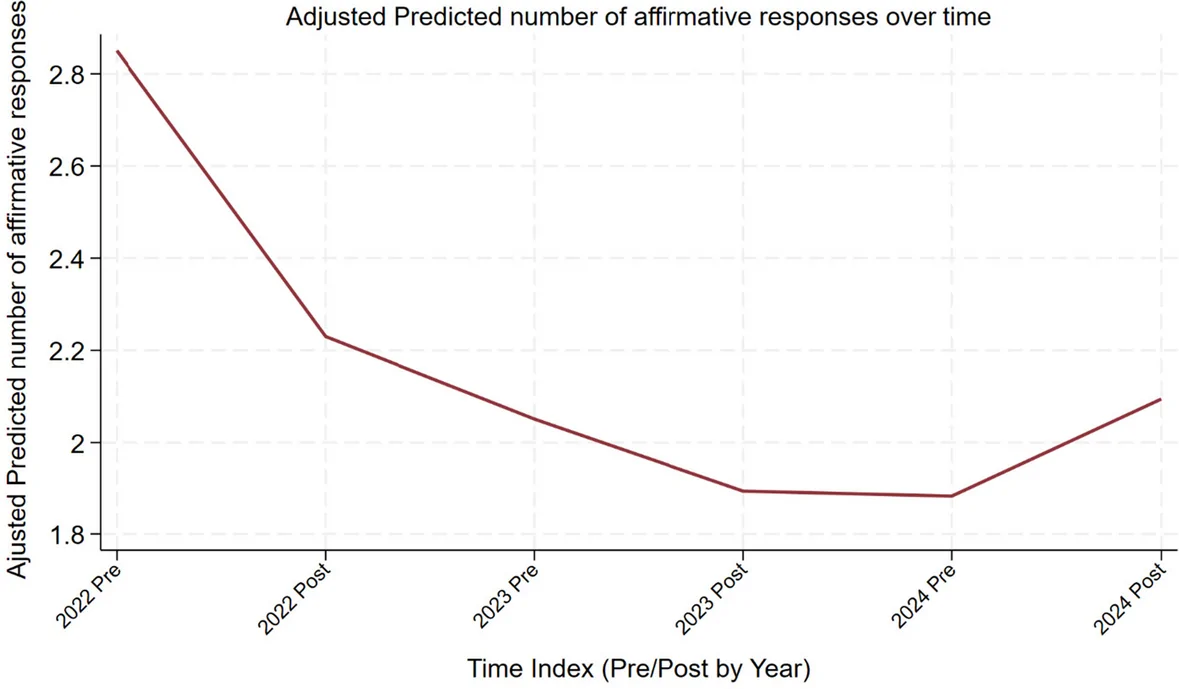
Longitudinal mixed effects model estimates of adjusted predicted number of affirmative responses across three program seasons

In longitudinal analyses*,* there was a significant reduction in the number of affirmative responses to HFSSM items through 2023, followed by attenuation in 2024. The adjusted mean number of affirmative responses for food security decreased from 2.85 (± 0.15) in the baseline to 1.88 (± 0.13) at the 2024 pre-intervention, then increased slightly to 2.09 (± 0.15). In mixed-effects models, the number of affirmative responses decreased by β = −0.75 (95% CI: −1.11 to −0.39) over three seasons (Table S5). Variance decomposition indicated that some 58% of the variability reflected within-person changes over time (Table S4). Figure 6 illustrates this pattern, showing a decline in the number of affirmative responses through 2023, followed by a modest increase at the final timepoint, which remained below the 2022 baseline.

In multilevel ordinal models, the odds of being in a more food-secure category increased in 2022 post (OR = 2.44, 95% CI 1.29 to 4.62) with continued improvements toward 2023 post-intervention(OR = 2.59, 95%CI 1.49 to 4.51), peaking in in 2024 pre-intervention (OR = 3.06, 95% CI 1.76 to 5.34) before attenuating in 2024 post-intervention (OR = 2.04, 95%CI 1.13 to 3.69)(Table 4).

**Table 4:** Multilevel ordinal Logistic regression of food security status over time

Time period	**Intervention**	Odds Ratio	Confidence Interval		***P*-value**	P-trend	Odds Ratio	Confidence Interval		***P*-value**	P-trend
		***Model 1***					***Model 2***				
2022	Pre					0.01					< 0.05
	Post	2.35	1.26	4.37	< 0.01		2.44	1.29	4.62	< 0.01	
2023	Pre	1.93	1.16	3.21	< 0.05		2.28	1.34	3.87	< 0.01	
	Post	2.38	1.40	4.05	< 0.01		2.59	1.49	4.51	< 0.001	
2024	Pre	2.61	1.54	4.43	< 0.001		3.06	1.76	5.34	< 0.001	
	Post	1.92	1.09	3.37	< 0.05		2.04	1.13	3.69	< 0.05	

Model 1: Unadjusted

Model 2 adjusted for age, gender, ethnicity, household size, presence of children under 17, employment status, insurance status, SNAP, and county

**Fig. 7:**
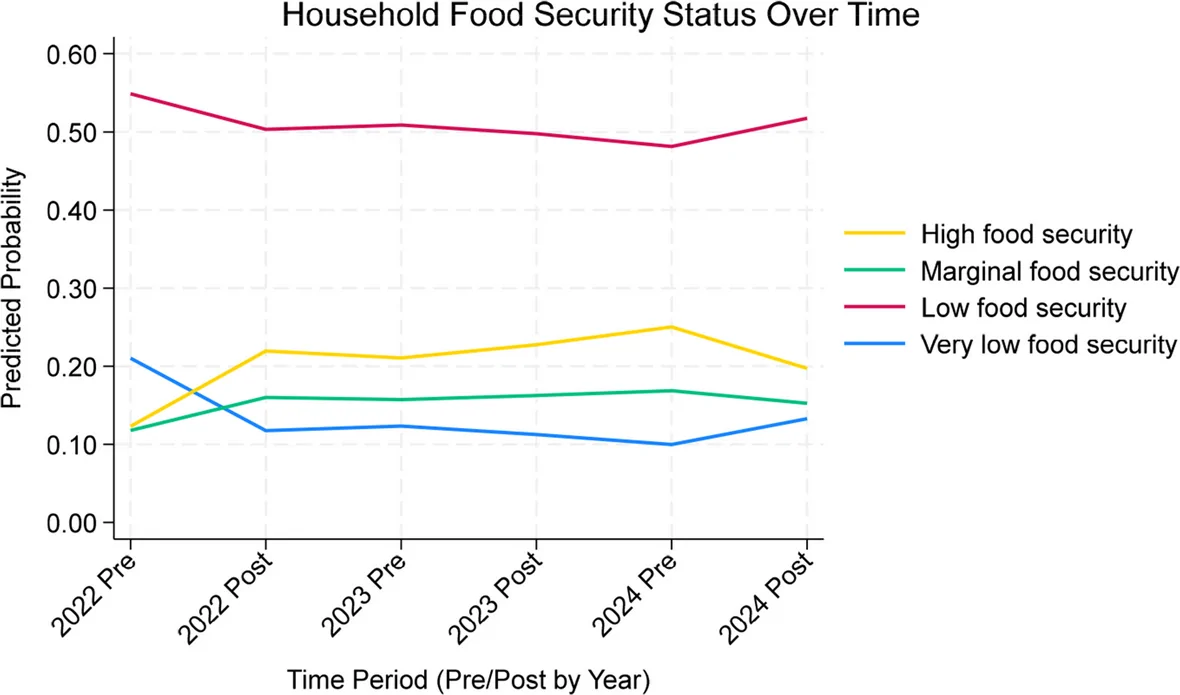
Household food security status over time: Predicted Probabilities from multilevel ordinal logistic regression

Adjusted predicted probabilities showed a redistribution toward higher food security categories. The probability of the very low food security category decreased from 21% at baseline to 11.7% post-intervention in 2022, then remained lower at 12.3% pre-intervention in 2023, 11.2% post-intervention in 2023, and 10% pre-intervention in 2024. Concurrently, high food security status increased from 12.3% to 25%, and marginal food security from 11.8% to 16.9% in 2024 pre-intervention. These improvements occurred alongside a persistent concentration in the low food security category, which remained the most prevalent across all time points at approximately 48–52%, as shown in Fig. 7 and Table S6. By 2024, some early gains were partially reversed: the probability of very low food security rose to 13.3%, while high food security declined to 19.7%, and low food security increased to 51.7%. Sixty-six percent of the variability in food security status was attributable to within-person changes over time (ICC = 0.34) (Table S4).

#### Diet quality perception

##### Year by year

In 2022, among all participants surveyed before and after the intervention, 36.5% rated their health as “Fair”, and 13.1% as “Poor.” These ratings slightly decreased post-survey to 32.02% and 5.23%, respectively. Among matched participants, 30.77% reported an improvement in perceived diet quality. Beyond perceived diet quality, 84.4% of respondents across all post-surveys reported learning new things about their diet. However, no statistically significant changes were observed between or within participants (all *p* > 0.05), despite some participants reporting improvements in perceived diet quality (Table not shown).

In 2023, at enrollment, 39.54% rated their health as “Fair” and 11.22% as "Poor," decreasing to 32.49% and 5.68% by the end of the season. No significant difference was observed between participants (Rank-sum test, *p* > 0.05). Among matched participants, 34.27% reported an improvement in perceived diet quality by at least one category, while 16.53% reported a decline with a significant shift in category distribution (Stuart-Maxwell test, *p* < *0.01*; Table 5).

**Table 5:** Changes in self-rated diet quality among matched participants (2023)

Diet Post → Diet Pre↓	Poor N(%)	Fair N(%)	Good N(%)	Very good N(%)	Excellent N(%)	Total N(%)
Poor	7(25)	14(50)	6(21)	1(3.5)	0(0)	28(100)
Fair	6(6.45)	45(48.3)	34(36.5)	5(5.37)	3(3.2)	93(100)
Good	1(1.0)	23(23.7)	53(54.6)	17(17.5)	3(3.1)	97(100)
Very good	0(0.0)	1(3.7)	9(33.3)	15(55.5)	2(7.4)	27(100)
Excellent	0(0.0)	0(0.0)	1(33.3)	0(0.0)	2(66.6)	3(100)
Total	14(5.6)	83(33.5)	103(41.5)	38(15.3)	10(4.0)	248(100)

In 2024, 39.82% rated their diet as “Fair” or “Poor” (10.74%) at enrollment, decreasing to 32.47% and 5.63% after the intervention. Among matched participants, 26.73% reported improved diet quality, while 18.32% reported a decline. No significant change was observed between or within participants (all *p* > 0.05) (Table not shown).

#### Changes in Diet quality perception over time:

**Fig. 8:**
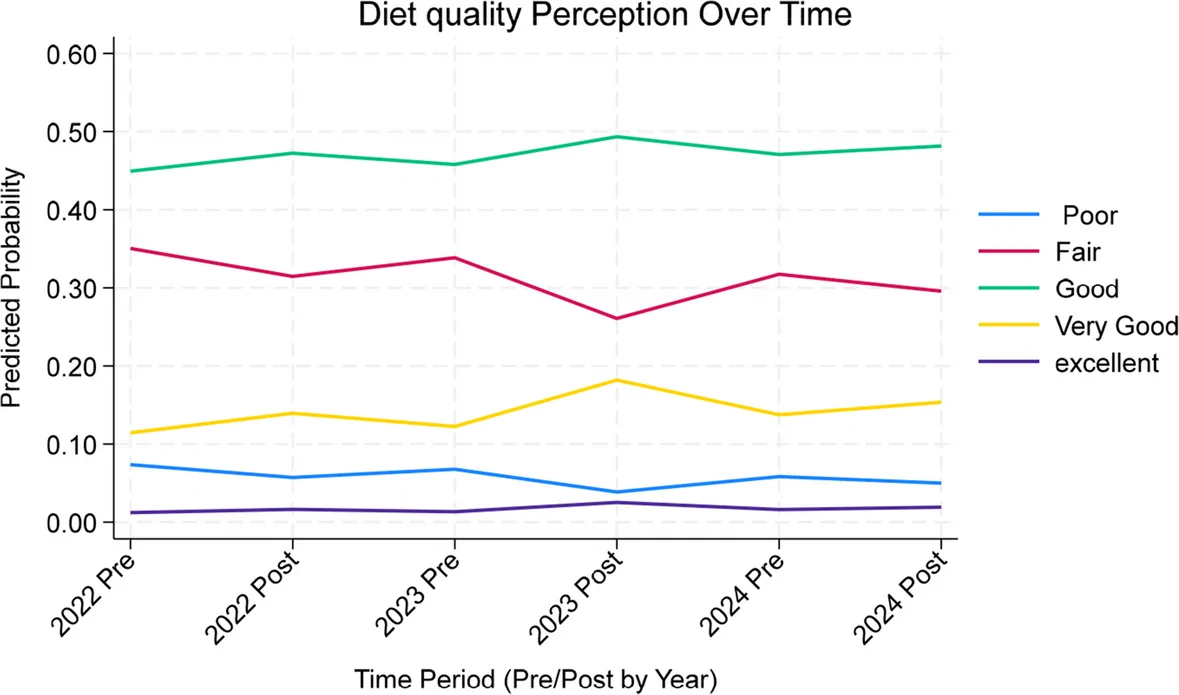
Diet Quality Perception Over time: Predicted Probabilities from multilevel ordinal logistic regression

Diet quality perception improved over time, although the effects were not consistent across all time points. Marginal predicted probability showed consistent post-program shifts towards higher diet-quality categories, with a reduction in “Poor/Fair” and increases in “Good”, “Very Good”, and “Excellent” ratings (Fig. 8). The largest improvement occurred in 2023, where the probability of reporting “Very Good” diet quality increased from 12.3% pre-intervention to 18% post-intervention. Adjusted odds ratios support these patterns. Relative to baseline, only the 2023 post-program showed a significant improvement in perceived diet quality (OR = 2.43, 95% CI 1.39–4.27), indicating more than double the odds of reporting higher diet quality (Table 6). No statistically significant difference was observed at other time points. The overall time *p-trend* suggested a significant gradual upward shift in perceived diet quality across the intervention seasons.

**Table 6:** Multilevel ordinal Logistic regression of diet quality ratings over time

Time period	Intervention	Odds Ratio	Confidence Interval		***P***-value	P-trend	Odds Ratio	Confidence Interval		***P***-value	P-trend
		***Model 1***					***Model 2***				
2022	Pre					0.01					< 0.05
	Post	1.51	0.83	2.75	0.17		1.43	0.78	2.64	0.25	
2023	Pre	1.10	0.66	1.84	0.70		1.13	0.67	1.92	0.64	
	Post	2.37	1.38	4.07	< 0.01		2.43	1.39	4.27	< 0.01	
2024	Pre	1.50	0.88	2.54	0.13		1.39	0.80	2.42	0.23	
	Post	1.95	1.10	3.45	< 0.05		1.72	0.95	3.12	0.07	

Model 1: Unadjusted

Model 2 adjusted for age, gender, ethnicity, household size, presence of children under 17, employment status, insurance status, SNAP, and county

### Self-rated health status

In 2022, 43.3% of participants reported “Fair” (43.3%) and “Poor” (10.5%) health status at enrollment, which decreased slightly to 37.08% and 9.27% poor health. Overall health status did not differ significantly between participants surveyed before and after the intervention (Rank-sum test, *p* > 0.05). Within matched participants, 25.55% improved by at least one category while 14.03% declined. However, the Stuart-Maxwell test indicated no significant shift in self-reported health category distribution within participants (*p* > 0.05).

In 2023, 40.49% reported “Fair” and 13.69% “Poor” at enrollment, decreasing to 33.22% and 12.66% by the end of the season. Between participants, no significant difference in health status was observed (Rank-sum test *p* > 0.05). Among matched participants, 25.8% reported a health improvement, while 15.72% reported a decline; the Stuart-Maxwell test indicated a statistically significant within-person shift in category distribution (*p* < 0.05; Table 7).

**Table 7:** Changes in self-rated health status among matched participants (2023)

Health Post → Health Pre ↓	Poor N(%)	Fair N(%)	Good N(%)	Very good N(%)	Excellent N(%)	Total N(%)
Poor	19(52.8)	14(39)	3(8.3)	0(0.0)	0(0.0)	36(100)
Fair	13(13.0)	53(53.0)	30(30.0)	2(2.0)	2(2.0)	100(100)
Good	0(0.0)	12(14.8)	57(70.4)	12(14.8)	0(0.0)	81(100)
Very Good	1(4.0)	0(0.0)	11(44.0)	12(48.0)	1(4.0)	25(100)
Excellent	0(0.0)	1(16.7)	1(16.7)	0(0.0)	4(66.6)	6(100)
Total	33(13.3)	80(32.2)	102(41.1)	26(10.5)	7(2.8)	248(100)

In 2024, overall “fair” and “poor” ratings were 38.17% and 12.95%, respectively, decreasing to 34.20% and 8.65% by the end of the season. Among matched participants, 20.97% reported improved health, and 21.95% reported a decline. No statistically significant changes were observed between or within participants (all *p* > 0.05). No significant changes in self-reported health status were observed over time (results not shown).

#### Program engagement

In 2023 and 2024, voucher redemption data indicated consistently high program engagement among participants. Nearly all participants, 91.41% (2023) and 96.17% (2024), received vouchers during the seasons. On average, about 65.3% redeemed their benefits more than 10 times, while just one quarter (27.1%) redeemed them between 3 and 10 times (Table S7).

#### Behavioral changes

Across all three years, a majority of respondents reported positive shopping practices, including spending more time in the produce section, feeling more confident while doing so, and experiencing less stress or worrying less during shopping (Table S8). The provision of F&V using vouchers appeared to motivate purchasing behaviors: most participants reported buying more produce, particularly items they consumed regularly, and many have expanded their choices to include varieties they don’t usually purchase. A few reported buying fruits and vegetables they had never eaten before.

#### Program perceptions—insights from participants' feedback

Via the post-survey, participants were also invited to share feedback. A total of 581 responses were analyzed, and key benefits of the program were reported as access to fruits and vegetables (26.8% of responses) and affordability **(**22.5% of responses**)**. Respondents also valued the program’s contribution to their health and nutrition (14.3% of responses), often noting improvement in diet quality or increased motivation to make healthier choices. Other commonly mentioned aspects included the freshness and quality of the produce (12.6% of responses), as well as variety and exploration (9.9% of responses). Additional aspects included access to cooking and nutrition education (4.5%), the program’s support for household food needs (2.6% of responses), and its ease of use (1.5% of responses).

#### Program perceptions—areas for growth

A total of 405 open-ended responses were analyzed across all program years to assess participants' suggestions for improving the PRx program. Almost half of the suggestions included expanding the vendor network (48.9%), increasing the voucher amount (14.1%), and extending the program duration to year-round (5.2%). Other suggestions included food variety and inclusion (4.2%), offering more cooking and nutrition education classes (3.7%), and improving the ease of using vouchers (3.2%). Participants also noted the need to improve store acceptance (3.2%) and requested smaller vouchers (e.g., $1 increments) to improve voucher usability by expanding retailer acceptance and offering flexible denominations (2.7%) for more flexible use. Other suggestions, including transportation support, implementation of a card system or more payment options, vendor education and training, and extending the program’s geographic reach, were also noted.

## Discussion

This study provides a comprehensive evaluation of the PRx program and provides evidence that a multi-year produce prescription model can yield sustained improvements in fruit and vegetable Intake and food security among rural populations, despite limited and inconsistent changes in diet and health perceptions. While prior evaluations of PRx programs have been mainly limited to single-season or cross-sectional assessments, our findings extend the literature by capturing within-person changes over multiple years and examining a broader set of outcomes. The use of mixed-effects modeling enabled us to account for individual-level variation, thereby reinforcing the robustness of these effects. By employing both year-by-year pre-post and longitudinal analyses, we capture both immediate impacts and long-term impacts, methods increasingly recognized as critical in “food as medicine” evaluations.

Over successive seasons, participants showed significant increases in fruit and vegetable intake, with evidence of both short-term gains and cumulative increases. Year-by-Year analyses showed significant within-season improvements in 2022 and to a lesser extent in 2024, with effect sizes ranging from small to moderate. Our findings are consistent with those of other seasonal nutrition incentive programs that reported higher intake of fruits and vegetables post-intervention [35, 36]. Similarly, Ralston et al. found a mean increase of 2.9 times/day in combined fruit and vegetable intake post-intervention [37], while Wilcox et al. reported a small increase in fruits and vegetables post-intervention (d = 0.17) [38]. Longitudinal mixed-effects models also showed consistent upward trends across all F&V categories over the three seasons. When considered alongside findings from prior interventions, these results suggest that immediate dietary changes vary across program seasons and that repeated exposure to the intervention contributes to gradual, cumulative improvements over time. This interpretation is further supported by the predominance of the within-person variability, indicating that observed changes reflect behavioral shifts within individuals. Overall, these findings emphasize the importance of sustained engagement in promoting dietary behavior change, suggesting that multi-year participation yields greater benefits than a single-season exposure.

In parallel, participants showed an increase in fried potato intake in 2022, despite reporting gains in dietary knowledge, followed by a reduction in 2024. This pattern may reflect a transitional phase in dietary behavior, characterized by an increase in “produce adjacent” items or a substitution toward familiar staples before more health-conscious substitutions take hold. Transtheoretical models applied to dietary behavior have documented that changes toward healthy eating unfold in a non-linear manner through discrete stages, where individuals remain in earlier phases, where familiar staples are modified before progressing into action and maintenance [37, 38]. Within this framework, the initial increase in fried potato intake may represent an intermediate step in dietary adaptation, while subsequent decline may signal the transition towards healthier food choices as knowledge, skills, and exposure to the intervention accumulate over time.

Self-reported diet quality improved directionally across all three intervention years, with consistent post-program reduction in “poor” and “fair” ratings and corresponding increases in “good” and “very good” ratings. However, these improvements were inconsistent. These patterns are not unexpected given that perceived diet quality is a subjective construct influenced not only by actual dietary intake but also by nutrition knowledge, expectations, and personal beliefs about what constitutes a healthy diet. As a result, directional improvements in intake may not always translate into measurable shifts in self-perception. Although fruit and vegetable intake increases significantly across all seasons in our mixed-effect models, participants may not interpret these changes as sufficient to shift their overall diet quality rating. Prior studies have demonstrated that self-rated diet quality is associated with healthier dietary patterns [39, 40], yet a large body of evidence also shows that many U.S. adults misjudge their diet quality, with substantial proportions often overestimating or inaccurately rating it [39, 40]. The pattern observed in our study aligns with existing literature suggesting that PRx program participation may nudge perception of diet quality upward, but subjective assessments remain variable and may not respond uniformly to intervention exposure.

Changes in self-rated health status were more limited across program years. Although small reductions in “fair” and “poor” health status were observed each season, some improvements were comparable across participants reporting worsened health status**.** Fruit and vegetable intake improvements did not translate into improved general health status. This may be due to a high proportion of participants unable to work and living with chronic conditions, factors that could blunt near-term shifts in perceived health even as diet improves. Moreover, the subjective nature of self-reported health assessment may not fully capture incremental changes in one’s overall well-being. These mixed findings may also reflect a lag between dietary changes and perceived or actual health improvements, particularly among individuals managing chronic disease, as persistent stressors and comorbidities may continue to influence health perceptions, even as food access improves.

Household food security improved over time with evidence of both within-season gains and cumulative improvements across repeated program exposure. Within-year gains showed moderate reductions in the number of affirmative responses to the HFSSM, alongside transitions mainly towards improved food security status. This pattern mirrors prior reports that PRx programs and related nutrition incentive strategies can lead to immediate gains and improve food security by alleviating economic constraints on purchasing healthy food [41]. The provision of no-cost produce reduces the financial burden of healthy eating. Enabling households to use vouchers to purchase fruits and vegetables facilitates access and affordability and reduces food-related financial stress [36]. The longitudinal models reinforced these findings, demonstrating a cumulative reduction of food insecurity, with more than half of the variability attributable to individual-level changes. The number of affirmative responses to HFSSM consistently decreased, and the odds of being in a more food-secure category increased across all time points, with predicted probabilities showing a redistribution away from very low, low, and marginal food security towards higher categories.

There has been increasing evidence suggesting the need for long-term assessment of the PRx program's impact on food security. A recent multi-year cross-sectional study found that food security status improves with longer participation in Nutrition Incentive projects [35]. However, this study faced limitations in evaluating project impact over time. Our findings add important nuance to the growing literature on food-as-medicine interventions, highlighting the critical role of sustained financial relief in improving access to and affordability of healthy foods, thereby reducing the severity of food insecurity.

Despite these improvements, food insecurity remained prevalent across all time points, affecting approximately half of the participants. This persistent concentration highlights that while the intervention reduced the severity of food insecurity, it did not fully transition households into food security. The continued high probability of being food insecure post-intervention underscores the influence of broader structural determinants that may not be fully addressed by food-based interventions alone.

The PRx program initiative also revealed behavioral shifts that extended beyond increases in fruit and vegetable Intake. The program demonstrated high participant retention, strong acceptability, and feasibility, evidenced by high voucher redemption and noticeable changes in shopping habits and produce choices sparked by voucher availability. By reducing the financial barrier to acquiring fresh produce, the program appeared to reframe grocery shopping from a constrained, stressful necessity into a more confident and less stressful experience. Participants not only spent more time in the produce section but also approached selection with increased confidence. Many reported trying new produce, including items they had never consumed. This may reflect greater openness to dietary variety, improved nutrition knowledge, or cooking skills [42]. Changes in shopping and purchasing behaviors likely stem from a combination of improved affordability and access to fresh produce. These patterns mirror prior findings that economic incentives can catalyze healthier purchasing behaviors through both financial and psychological experiences of shopping [43–45].

### Program perceptions

In addition to observed behavioral changes, participants’ feedback provided valuable insight into how the program was experienced and perceived. The most notable benefits were the access and affordability of F&V, along with the Freshness of the produce and reported gains in health and nutrition. These responses indicate that participants valued both the instrumental aspects (cost, access) and the quality of the produce offered. While not central to most participants’ experiences, mentions of produce variety, cooking, and nutrition education suggest that these components may enrich and reinforce dietary behavior change [46, 47]. Despite the overall positive reception of PRx program, participants highlighted some barriers and challenges faced during the program. These suggestions were pragmatic and revealed systemic opportunities for enhancing impact.

#### Expand collaboration with more stores/vendors

While the PRx program effectively improved access to fruits and vegetables, its impact was partially constrained by the geographic distribution and accessibility of participating retailers. This challenge has been well documented in food access research, which notes that people tend to make food choices based on the food outlets available in their immediate neighborhood. Therefore, limited vendor availability can reduce both the frequency and variety of purchases [48, 49]. Given that our results demonstrated both short- and long-term gains in fruit and vegetable intake and food security, expanding the vendor network could amplify these effects by enabling more consistent and diverse purchasing over time.

#### Increasing the voucher amount

The PRx Program provides approximately $15/week in produce vouchers to individual enrollees. However, many participants reported living in households of two to three people, which may limit the overall dietary impact of the benefit. Compared to other programs reviewed, the PRx program offers a relatively high voucher value/week. Yet, some studies reported providing more than $15/week and/or scaling amounts based on household size, with weekly values ranging from $5 to $75 [17, 18, 21, 50, 51]. These models suggest that tailoring voucher amounts to reflect household composition may improve dietary impact and participant satisfaction.

#### Extending the program’s duration to operate year-round

Participants suggested extending the program to operate year-round. Seasonal models may lead to regression in dietary behaviors and food security when financial support ends. A year-round model could help support long-term adoption of dietary behaviors and food security gains, reducing the risk of relapse and reinforcing the integration of fresh produce into routine dietary habits.

#### Increasing the variety of produce offered and expanding the types of foods eligible for purchase

While the program already encouraged dietary diversification, as evidenced by participants’ reports of trying unfamiliar fruits and vegetables, further broadening the selection could sustain engagement and prevent “menu fatigue”. Dietary variety is associated with higher overall diet quality, improved micronutrient intake, and better long-term health outcomes [52, 53]. Expanding product eligibility to include “whole grains, “milk, " eggs, and other healthy, culturally relevant items could enhance inclusivity and increase program uptake.

#### More cooking and nutrition education classes

This suggestion reflects an interest in skill-building alongside financial assistance. Existing literature suggests that the synergistic benefits of combining nutrition incentives with education can enhance confidence in preparing food, reduce waste, and strengthen the link between access and actual consumption [54]. Translating access into sustained healthy eating practices may require complementary education, as shown through participants’ increased confidence and exploratory behavior in their shopping.

#### Improving voucher usability

Requests for a smaller voucher reflected a desire for greater control over benefit use, enabling multiple smaller transactions instead of fewer large ones. Improving voucher acceptance through expanded retailer participation and simplified point-of-sale processes would ensure that the intervention reaches its full potential.

In addition to commonly mentioned suggestions, other, less frequently cited but equally important suggestions emerged. These included transportation support, the implementation of card-based payment, vendor education and training, and geographic expansion of the program. Each aligns with broader determinants of food access. For instance, transportation remains a significant barrier for low-income individuals striving to maintain a healthy diet [55]. Even when vendors are available, limited transportation can limit redemption. Introducing modern payment methods, such as card-based systems, could reduce friction at the point of sale, improve convenience, and enable better tracking of program use. Vendor training on PRx program goals and procedures can ensure smooth interactions, reduce stigma, and potentially increase redemption rates.

### Strengths and limitations

A key strength of this evaluation is its year-by-year and longitudinal design, which enabled assessment of both immediate and long-term impacts across multiple seasons. This addresses a significant gap in most produce prescription evaluations, which typically rely on short-term, single-season data. Additionally, integrating quantitative and qualitative data provided a comprehensive understanding of program outcomes and the contextual factors influencing impact. However, several limitations should be acknowledged. These include self-reported measures, which may be affected by recall bias; the lack of a control group; the program's seasonal nature; and potential limitations in generalizing findings to other clinical settings. Because program eligibility was determined through healthcare provider referral, individuals without access to health care (e.g., those facing structural barriers such as transportation, cost, or lack of insurance) were excluded, potentially limiting the program's reach. Furthermore, the lack of cardiometabolic biomarker data limits the ability to evaluate physiological changes following the intervention. Despite these limitations, this evaluation provides essential insights for future program refinements. Our findings add to the growing evidence supporting produce prescription programs as a scalable intervention to address nutrition-related health disparities, especially in rural communities with limited access to food; however, because intake in this study reflects frequency rather than quantity, observed improvements should be interpreted as shifts in eating patterns rather than absolute increases in dietary intake.

### Public health implications

By offsetting some food costs and supporting healthier dietary behaviors, the PRx program was associated with modest increases in produce consumption and reductions in the severity of household food insecurity. Our findings are significant given the strong connection between diet quality, food insecurity, and chronic disease risk. However, changes in self-rated health were inconsistent, suggesting that the impact on perceived overall health may be limited. Participant recommendations provide a roadmap for enhancing reach and impact. As health systems increasingly explore “food as medicine” interventions, incorporating PRx program models into routine care could be a cost-effective, community-centered approach to improving diet quality and reducing the burden of diet-related chronic diseases in the U.S.

## Conclusion

The evaluation of the PRx Program showed that participation was associated with improvements in fruit and vegetable Intake and food insecurity severity among rural, medically underserved populations with diet-related chronic diseases. Sustained participation across multiple seasons was associated with consistent dietary gains and reductions in food insecurity severity, highlighting the potential for “food as medicine” strategies to support both short-term and long-term benefits. While improvements in fruit and vegetable intake were clear, changes in self-rated health and perceived diet quality were more limited and inconsistent. Overall, the PRx program was well-received and demonstrated the potential to support improvements in dietary behaviors and food insecurity severity over repeated program exposure. Its public health impact may be strengthened by integrating structural and cultural adaptations informed by participant feedback.

Our findings underscore three key takeaways:

1. sustained engagement improves dietary behaviors and reduces food insecurity severity,
2. self-rated health improvements may lag behind behavioral changes and
3. participants' experiences and structural context are critical to program impact and long-term scalability.

## Supplementary Information

Supplementary Material 1.

Supplementary Material 2.

Supplementary Material 3.

## Data Availability

The data supporting the findings of this study are available from the corresponding author upon request. Access is subject to a data use agreement and approval from the Institutional Review Board (IRB).

## Abbreviations

BIPOC: Black Indigenous, and People of Color
CBO: Community-based organization
CSA: Community Supported Agriculture
DSQ: The Dietary Screener Questionnaire
EFNEP: Expanded Food and Nutrition Education Program
FI: Food Insecurity
FS: Food Security
F&V: Fruits and Vegetables
FVI: Fruit and Vegetable Intake
GusNIP: Gus Schumacher Nutrition Incentive Program
HHFSSM: Household Food Security Survey Module
ICC: Intraclass Correlation Coefficient
IRB: Institutional Review Board
NYS: New York State
PRx: Produce Prescription
PRxSCNY: Produce Prescription Program of South Central New York
SD: Stndard deviation
SNAP: Special Nutrition Assistance Program
WIC: Special Supplemental Nutrition Program for Women and Children
