# Supplementary material for: Evaluating the impact of a multi-year produce prescription program on fruit and vegetable intake, and food security in south central New York
Source: Int J Behav Nutr Phys Act. 2026 Jul 28;23:101. doi: 10.1186/s12966-026-01955-4 (PMC13692909; doi:10.1186/s12966-026-01955-4)
Supplement: Supplementary file 1 [file 12966_2026_1955_MOESM1_ESM.docx]

**PRxSCNY – Produce Prescription Program of South Central New York**

**Program design**

The PRxSCNY is managed and implemented by the Rural Health Network of South Central New York, a regional community-based organization (CBO) that helps address health-related social needs. In response to the dual crises of FI and diet-related chronic diseases, a pilot PRx intervention was designed and delivered through a partnership between health-care and social-services providers. The PRxSCNY was established in 2018 to help improve access to nutrient-rich foods, particularly for low-income families. Its programming includes outreach and support, access to education materials and programming, and free or subsidized fresh F&V.

The pilot began in Broome County, expanding to neighboring Delaware and Tioga counties in year two and Tompkins County in year three. The program aimed to increase food security among lower-income residents with diet-related chronic diseases (e.g., diabetes, pre-diabetes, cardiovascular disease, or BMI ≥30), and increase fruit and vegetable(F&V) consumption(1) Between 2017 and 2021, supported by local grant dollars, the program partnered with 11 clinical providers and served over 1000 households across three Counties.

In 2022, with support from the Gus Schumacher Nutrition Incentive Program (GusNIP), the PRxSCNY refined its model and expanded its partnerships with the aim of reaching 825 eligible participants across four rural counties in NYS’ Southern Tier. The goals of the expanded program aimed to build a sustainable PRx program network that improves patients’ dietary health, FS, and health outcomes, while becoming more fully integrated into healthcare practices. Program data were collected for three complete project years (2022, 2023, 2024). With program expansion, PRxSCNY expected to achieve four goals among participants: (1) 80% report an increase in F&V access, (2) 75% report an increase in F&V consumption, (3) 80% report a reduction in FI, and (4) 80% report an increase in self-perceived health.

**Implementation and participant engagement**

The PRx Program runs from mid-June to November. During this period, participants are identified, enrolled by their clinical providers, and prescribed produce vouchers or CSA farm shares. Participants may choose to receive a weekly CSA/farm share or receive $120 worth of vouchers at each of three clinic visits (up to $360 total for the season) (1). Patients who opt in to participate must connect with their clinical provider every two months to maintain access to vouchers or CSA coupons (given at an in-person visit, or sent via mail if a telemedicine visit).

Beyond clinical engagement, participants received continuous support from the non-clinical PRx staff via (e.g., private staff-monitored Facebook group, group nutrition classes, one-on-one support from community health workers or health coaches). Automated text and email reminders are also utilized to encourage voucher redemption, class attendance, return for healthcare visits, and complete surveys. Seasonal recipes, tips, encouragement, and invitations to join the community advisory committee with options for video or telephone participation are also provided.

To help participants overcome possible transportation barriers, PRxSCNY partnered with the Getthere program at Rural Health Network to provide mobility assistance to access medical appointments and shop with vouchers. The PRxSCNY also utilized several home-delivery options for CSA shares, including DoorDash and direct delivery from partner farms.

1.	Network FaH. Produce Prescription Program of South Central NY

Information for health care providers Binghamton, NY: Food and Health Network; 2025 [cited 2025 04/08]. Available from: <https://foodandhealthnetwork.org/prxproviders/>.
