# Supplementary material for: Evaluating the impact of a multi-year produce prescription program on fruit and vegetable intake, and food security in south central New York
Source: Int J Behav Nutr Phys Act. 2026 Jul 28;23:101. doi: 10.1186/s12966-026-01955-4 (PMC13692909; doi:10.1186/s12966-026-01955-4)
Supplement: Supplementary file 2 [file 12966_2026_1955_MOESM2_ESM.docx]

| **Table S1. Year-by-Year Independent Comparison of Daily Frequency Intake by Food Category before and after the program.** | | | | | | | | | | | | | |
| --- | --- | --- | --- | --- | --- | --- | --- | --- | --- | --- | --- | --- | --- |
|  |  | **2022** | | | | | | **2023** | | | | | |
| **Food Category** |  | **Median** | **Mean (SD)** | **N** | **Z** | **Effect Size** | **P-value** | **Median** | **Mean (SD)** | **N** | **Z** | **Effect Size** | **P-value** |
| Fruits | Pre | 0.50 | 0.57(±0.53) | 413 | -4.88 | 0.21 | <0.001 | 0.50 | 0.93(±1.05) | 514 | 2.18 | 0.07 | 0.02 |
|  | Post | 0.50 | 0.83(±0.62) | 153 |  |  |  | 0.78 | 0.93(±0.79) | 320 |  |  |  |
| Green Vegetables | Pre | 0.28 | 0.36(±0.39) | 413 | -2.81 | 0.12 | <0.01 | 0.28 | 0.51(±0.63) | 512 | 2.52 | 0.09 | 0.01 |
|  | Post | 0.28 | 0.43(±0.41) | 152 |  |  |  | 0.50 | 0.54(±0.52) | 316 |  |  |  |
| Fried Potatoes | Pre | 0.14 | 0.22(±0.28) | 414 | 0.37 | 0.02 | 0.71 | 0.08 | 0.15(±0.21) | 511 | 0.01 | 0.0005 | 0.98 |
|  | Post | 0.14 | 0.28(±0.31) | 153 |  |  |  | 0.08 | 0.17(±0.28) | 321 |  |  |  |
| Potatoes | Pre | 0.14 | 0.26(±0.30) | 414 | 0.62 | 0.03 | 0.53 | 0.14 | 0.24(±0.28) | 514 | 0.61 | 0.02 | 0.54 |
|  | Post | 0.14 | 0.28(±0.31) | 153 |  |  |  | 0.14 | 0.23(±0.23) | 322 |  |  |  |
| Other Vegetables | Pre | 0.50 | 0.53(±0.46) | 414 | -3.49 | 0.15 | <0.001 | 0.50 | 0.69(±0.65) | 513 | 2.18 | 0.07 | 0.02 |
|  | Post | 0.50 | 0.67(±0.50) | 153 |  |  |  | 0.50 | 0.73(±0.61) | 320 |  |  |  |
| Beans | Pre |  |  |  |  |  |  | 0.08 | 0.21(±0.33) | 445 | 0.52 | 0.02 | 0.60 |
|  | Post |  |  |  |  |  |  | 0.08 | 0.21(±0.30) | 280 |  |  |  |
| Pizza | Pre |  |  |  |  |  |  | 0.03 | 0.10(±0.18) | 431 | -1.54 | 0.58 | 0.12 |
|  | Post |  |  |  |  |  |  | 0.03 | 0.08(±0.18) | 280 |  |  |  |
| Tomato sauce | Pre |  |  |  |  |  |  | 0.03 | 0.16(±0.18) | 401 | 0.52 | 0.02 | 0.60 |
|  | Post |  |  |  |  |  |  | 0.03 | 0.18(±0.27) | 269 |  |  |  |
| Fruit Juice | Pre |  |  |  |  |  |  | 0.08 | 1.02(±1.37) | 476 | 1.43 | 0.05 | 0.13 |
|  | Post |  |  |  |  |  |  | 0.43 | 1.22(±1.49) | 308 |  |  |  |
| Salsa | Pre |  |  |  |  |  |  | 0.03 | 0.09(±0.23) | 469 | -2.00 | 0.07 | 0.04 |
|  | Post |  |  |  |  |  |  | 0.00 | 0.09(±0.29) | 287 |  |  |  |

| **Table S1. Year-by-Year Independent Comparison of Daily Frequency Intake by Food Category before and after the program(continued).** | | | | | | | |
| --- | --- | --- | --- | --- | --- | --- | --- |
|  |  | **2024** | | | | | |
| **Food Category** |  | **Median** | **Mean (SD)** | **N** | **Z** | **Effect Size** | **P-value** |
| Fruits | Pre | 0.50 | 0.98(±1.10) | 440 | 2.03 | 0.07 | 0.04 |
|  | Post | 0.78 | 1.01(±0.85) | 232 |  |  |  |
| Green Vegetables | Pre | 0.28 | 0.51(±0.60) | 444 | 2.21 | 0.08 | 0.02 |
|  | Post | 0.50 | 0.59(±0.62) | 231 |  |  |  |
| Fried Potatoes | Pre | 0.08 | 0.21(±0.36) | 449 | -1.17 | 0.04 | 0.24 |
|  | Post | 0.08 | 0.20(±0.36) | 233 |  |  |  |
| Potatoes | Pre | 0.14 | 0.24(±0.34) | 446 | 0.25 | 0.009 | 0.80 |
|  | Post | 0.14 | 0.24(±0.33) | 232 |  |  |  |
| Other Vegetables | Pre | 0.50 | 0.74(±0.71) | 451 | 1.69 | 0.06 | 0.09 |
|  | Post | 0.50 | 0.78(±0.67) | 232 |  |  |  |
| Beans | Pre | 0.08 | 0.21(±0.38) | 394 | 0.25 | 0.01 | 0.80 |
|  | Post | 0.08 | 0.19(±0.28) | 205 |  |  |  |
| Pizza | Pre | 0.08 | 0.09(±0.17) | 392 | -2.14 | 0.09 | 0.03 |
|  | Post | 0.03 | 0.10(±0.27) | 195 |  |  |  |
| Tomato sauce | Pre | 0.08 | 0.17(±0.25) | 373 | 0.49 | 0.02 | 0.62 |
|  | Post | 0.08 | 0.19(±0.33) | 195 |  |  |  |
| Fruit Juice | Pre | 0.78 | 1.20(±1.45) | 425 | -0.28 | 0.01 | 0.78 |
|  | Post | 0.78 | 1.22(±1.46) | 220 |  |  |  |
| Salsa | Pre | 0 | 0.08(±0.23) | 413 | -0.74 | 0.03 | 0.45 |
|  | Post | 0.03 | 0.11(±0.33) | 208 |  |  |  |

| **Table S2: Mixed-effects regression estimates of long-term change in fruit and vegetable consumption(2022–2024)** | | | | | | | | | | | | | |
| --- | --- | --- | --- | --- | --- | --- | --- | --- | --- | --- | --- | --- | --- |
| **Foods** | **Intervention** | **ß** | **Mean(±SE)** | **Confidence Interval** | | **P-value** | **P-trend** | **Mean(±SE)** | **ß** | **Confidence Interval** | | **p-value** | **p-trend** |
|  |  |  | **Model 1** | | | |  |  |  | **Model 2** | | | |
| Fruits |  |  |  |  | |  | <0.01 |  |  |  |  |  | <0.01 |
| 2022 | Pre | Ref | 0.68(±0.07) | - | - | - |  | 0.66(±0.07) | - | - | - |  |  |
|  | Post | 0.12 | 0.80(±0.09) | -0.08 | 0.33 | 0.23 |  | 0.78(±0.90) | 0.11 | -0.09 | 0.32 | 0.29 |  |
| 2023 | Pre | 0.23 | 0.91(±0.06) | 0.06 | 0.40 | <0.01 |  | 0.94(±0.06) | 0.28 | 0.10 | 0.45 | < 0.01 |  |
|  | Post | 0.28 | 0.96(±0.07) | 0.10 | 0.45 | <0.01 |  | 0.98(±0.07) | 0.31 | 0.13 | 0.49 | <0.001 |  |
| 2024 | Pre | 0.29 | 0.96(±0.07) | 0.11 | 0.46 | <0.01 |  | 1.00(±0.07) | 0.34 | 0.16 | 0.52 | <0.001 |  |
|  | Post | 0.23 | 0.91(±0.08) | 0.04 | 0.42 | 0.02 |  | 0.93(±0.08) | 0.27 | 0.07 | 0.46 | < 0.01 |  |
| Fruit juice |  |  |  |  |  |  | >0.05 |  |  |  |  |  | <0.05 |
| 2023 | Pre | Ref | 1.07 (± 0.12) | - | - | - |  | 1.03(±0.11) | - | - | - |  |  |
|  | Post | 0.09 | 1.16 (± 0.13) | -0.19 | 0.36 | 0.54 |  | 1.16(±0.12) | 0.13 | -0.15 | 0.42 | 0.34 |  |
| 2024 | Pre | 0.11 | 1.18 (± 0.12) | -0.17 | 0.38 | 0.44 |  | 1.17(±0.12) | 0.14 | -0.14 | 0.42 | 0.32 |  |
|  | Post | 0.27 | 1.34 (± 0.14) | -0.04 | 0.57 | 0.08 |  | 1.36(±0.13) | 0.33 | 0.03 | 0.64 | <0.05 |  |
| Green vegetables |  |  |  |  |  |  | <0.01 |  |  |  |  |  | <0.01 |
| 2022 | Pre | Ref | 0.38 (± 0.04) | - | - | - |  | 0.38(±0.04) | - | - | - |  |  |
|  | Post | 0.03 | 0.42 (± 0.05) | -0.09 | 0.15 | 0.61 |  | 0.42(±0.05) | 0.04 | -0.08 | 0.16 | 0.48 |  |
| 2023 | Pre | 0.09 | 0.47 (± 0.04) | -0.01 | 0.19 | 0.09 |  | 0.48(±0.04) | 0.10 | -0.008 | 0.20 | 0.07 |  |
|  | Post | 0.13 | 0.52 (± 0.04) | 0.02 | 0.24 | 0.01 |  | 0.52(±0.04) | 0.13 | 0.02 | 0.24 | <0.05 |  |
| 2024 | Pre | 0.15 | 0.54 (± 0.04) | 0.05 | 0.26 | <0.01 |  | 0.55(±0.04) | 0.17 | 0.06 | 0.27 | < 0.01 |  |
|  | Post | 0.11 | 0.49 (± 0.05) | -0.007 | 0.22 | 0.06 |  | 0.49(±0.05) | 0.11 | -0.007 | 0.22 | 0.06 |  |
| Other vegetables |  |  |  |  |  |  | <0.01 |  |  |  |  |  | <0.01 |
| 2022 | Pre | Ref | 0.55 (± 0.05) | - | - | - |  | 0.56(±0.05) | - | - | - |  |  |
|  | Post | 0.11 | 0.67 (± 0.07) | -0.04 | 0.26 | 0.13 |  | 0.66(±0.07) | 0.09 | -0.05 | 0.25 |  |  |
| 2023 | Pre | 0.17 | 0.72 (± 0.05) | 0.04 | 0.29 | < 0.01 |  | 0.74(±0.05) | 0.18 | 0.05 | 0.30 | <0.01 |  |
|  | Post | 0.18 | 0.73 (± 0.05) | 0.05 | 0.31 | <0.01 |  | 0.73(±0.05) | 0.17 | 0.03 | 0.31 | <0.05 |  |
| 2024 | Pre | 0.16 | 0.72 (± 0.05) | 0.03 | 0.29 | 0.01 |  | 0.73(±0.05) | 0.17 | 0.04 | 0.30 | <0.05 |  |
|  | Post | 0.26 | 0.81 (± 0.06) | 0.12 | 0.40 | <0.001 |  | 0.82(±0.06) | 0.26 | 0.11 | 0.40 | <0.001 |  |

Model 1: Unadjusted

Model 2 adjusted for age, gender, ethnicity, household size, presence of children under 17, employment status, insurance status, SNAP, and county.

| **Table S3**: Food security status transitions among matched participants (2024) | | | | | |
| --- | --- | --- | --- | --- | --- |
| FS status Post →  FS status Pre↓ | High N(%) | Marginal N(%) | Low N(%) | Very Low N(%) | Total N(%) |
| High | 18(35.3) | 12(23.5) | 18(35.3) | 3(5.9) | 51(100) |
| Marginal | 10(30.3) | 8(24.2) | 14(42.4) | 1(3.0) | 33(100) |
| Low | 11(10.7) | 16(15.5) | 74(71.8) | 2(1.9) | 103(100) |
| Very Low | 3(13.6) | 2(9.1) | 6(27.3) | 11(50) | 22(100) |
| Total | 42(20.1) | 38(18.2) | 112(53.6) | 17(8.1) | 209(100) |

| **Table S4:** Between -and within-person variance estimates and Intra-Class Correlation (ICC) for daily FVI and Food security. | | | |
| --- | --- | --- | --- |
|  | **Variance estimates** | |  |
| **Unadjusted** |  | |  |
| **Foods** | **Within person** | **Between person** | **ICC** |
| Fruits | 0.48 | 0.20 | 0.30 |
| Fruit juice | 1.31 | 0.77 | 0.37 |
| Green vegetables | 0.17 | 0.08 | 0.33 |
| Other vegetables | 0.26 | 0.11 | 0.30 |
| Number of affirmative responses | 1.60 | 1.44 | 0.47 |
| Food security levels | - | 1.92 | 0.37 |
| **Adjusted** |  |  |  |
| **Foods** |  |  |  |
| Fruits | 0.49 | 0.16 | 0.24 |
| Fruit juice | 1.29 | 0.60 | 0.32 |
| Green vegetables | 0.17 | 0.06 | 0.26 |
| Other vegetables | 0.26 | 0.08 | 0.23 |
| Number of affirmative responses | 1.57 | 1.14 | 0.42 |
| Food security levels | - | 1.74 | 0.34 |

Model 1: Unadjusted

Model 2 adjusted for age, gender, ethnicity, household size, presence of children under 17, employment status, insurance status, SNAP, and county.

| **Table S5: Mixed-effects regression estimates of long-term change in number of affirmative responses for Food Security(2022–2024)** | | | | | | | | | | | | | | |
| --- | --- | --- | --- | --- | --- | --- | --- | --- | --- | --- | --- | --- | --- | --- |
| **Year** | **Food Security** | **Intervention** | **Mean (±SE)** | **ß** | **Confidence Interval** | | ***P-value*** | ***P-trend*** | **Mean (±SE)** | **ß** | **Confidence Interval** | | ***P-value*** | ***P-trend*** |
|  |  |  |  | ***Model 1*** | | | |  |  | ***Model 2*** | | | | |
| 2022 | Affirmations | Pre | 2.85(±0.15) | Ref | - | - | - | <0.0001 | 2.85(±0.15) |  |  |  |  | <0.0001 |
|  |  | Post | 2.22(±0.18) | -0.63 | -1.00 | -0.25 | 0.001 |  | 2.23(±0.18) | -0.62 | -0.99 | -0.24 | <0.01 |  |
| 2023 | Affirmations | Pre | 2.12(±0.13) | -0.73 | -1.04 | -0.42 | <0.001 |  | 2.05(±0.13) | -0.79 | -1.11 | -0.48 | <0.001 |  |
|  |  | Post | 1.89(±0.14) | -0.96 | -1.28 | -0.63 | <0.001 |  | 1.89(±0.14) | -0.95 | -1.29 | -0.62 | <0.001 |  |
| 2024 | Affirmations | Pre | 1.92(±0.14) | -0.92 | -1.24 | -0.60 | <0.001 |  | 1.88(±0.13) | -0.96 | -1.29 | -0.63 | <0.001 |  |
|  |  | Post | 2.09 (±0.16) | -0.76 | -1.10 | -0.41 | <0.001 |  | 2.09(±0.15) | -0.75 | -1.11 | -0.39 | <0.001 |  |

Model 1: Unadjusted

Model 2 adjusted for age, gender, ethnicity, household size, presence of children under 17, employment status, insurance status, SNAP, and county.

| Table S6: Predicted Probabilities from multilevel ordinal logistic regression with 95% confidence intervals. | | | | | |
| --- | --- | --- | --- | --- | --- |
| **Time period** | I**ntervention** | Food security status | | | |
|  |  | Very low | Low | Marginal | High |
| 2022 | Pre | 21.0 (14.8-27.4) | 54.9 (50.5-59.3) | 11.8 (8.8- 14.8) | 12.3 (7.9-16.7) |
|  | Post | 11.7 (6.8-16.7) | 50.3 (44.3-56.3) | 16.0 (12.6-19.5) | 21.9 (14.6-29.3) |
| 2023 | Pre | 12.3 (8.6-16.1) | 50.9 (46.2-55.6) | 15.7 (12.8-18.7) | 21.1 (15.9-26.2) |
|  | Post | 11.2 (7.5-15.0) | 49.8 (44.8-54.7) | 16.2 (13.2-19.3) | 22.7 (17.1-28.4) |
| 2024 | Pre | 10.0 (6.6-13.3) | 48.1 (43.0- 53.3) | 16.9 (13.8-19.9) | 25.0 (19.2-30.9) |
|  | Post | 13.3 (8.7-17.9) | 51.7 (46.8- 56.7) | 15.3 (12.1-18.4) | 19.7 (13.9-25.5) |
| Model adjusted for age, gender, ethnicity, household size, presence of children under 17, employment status, insurance status, SNAP, and county. | | | | | |

**Table S7: Benefits Redemption Frequency**

| USAGE FREQUENCY | 2023 (n = 318) | 2024 (n = 231) |
| --- | --- | --- |
| More Than 10 Times | 65.7% | 64.9% |
| 3 To 10 Times | 26.1% | 28.1% |
| 1 To 2 Times | 5.3% | 3.9% |
| Never Used | 2.8% | 3.0% |

**Table S8: Shopping and Purchasing Behaviors**

|  | 2022 | | 2023 | | 2024 | |
| --- | --- | --- | --- | --- | --- | --- |
| Shopping and purchasing behaviors | N | Percent (%) | N | Percent (%) | N | Percent (%) |
| SHOPPING BEHAVIORS |  |  |  |  |  |  |
| I spent more time in the produce section | 94 | 61.04 | 215 | 71.67 | 125 | 54.59 |
| I felt more confident | 74 | 48.05 | 116 | 38.67 | 89 | 38.86 |
| I felt less stressed or worried | 69 | 44.81 | 128 | 42.67 | 86 | 37.55 |
| PURCHASING BEHAVIORS |  |  |  |  |  |  |
| Bought more of the kinds I usually purchase | 103 | 69.59 | 170 | 71.43 | 149 | 62.60 |
| Bought kinds that I usually don’t purchase | 89 | 60.14 | 144 | 60.50 | 124 | 52.10 |
| Bought kinds I have never eaten before | 62 | 41.89 | 76 | 31.93 | 75 | 31.51 |
