# Supplementary material for: Evaluating the impact of a multi-year produce prescription program on fruit and vegetable intake, and food security in south central New York
Source: Int J Behav Nutr Phys Act. 2026 Jul 28;23:101. doi: 10.1186/s12966-026-01955-4 (PMC13692909; doi:10.1186/s12966-026-01955-4)
Supplement: Supplementary file 3 [file 12966_2026_1955_MOESM3_ESM.docx]

**The TIDieR (Template for Intervention Description and Replication) Checklist*:**

Information to include when describing an intervention and the location of the information

| **Item number** | **Item** | **Where located **** | |
| --- | --- | --- | --- |
|  |  | Primary paper  (page or appendix  number) | Other ^†^ (details) |
|  | **BRIEF NAME** |  |  |
| **1.** | Produce Prescription Program of South Central New York | Title Page | N/A |
|  | **WHY** |  |  |
| **2.** | Aims to improve access and intake of fruits and vegetables, improve Food Security, and health among low-income participants with diet-related chronic diseases | Page 5 | N/A |
|  | **WHAT** |  |  |
| **3.** | Produce vouchers/ CSA farm shares were prescribed by a health care provider to eligible participants. Participants choose to receive a weekly Community Supported Agriculture (CSA) share or receive $120 worth of vouchers at each of three clinic visits (up to $360 total for the season). Vouchers are redeemable at the local farmers’ market and participating retailers; farm shares are delivered or picked up at the farm. | Page 7 | Supplementary file 1 |
| **4.** | Participants are screened and referred to the program by their health care providers. Patients who opt in to participate must connect with their clinical provider every two months to maintain access to vouchers or CSA coupons (given at an in-person visit, or sent via mail if a telemedicine visit).  Beyond clinical engagement, participants received continuous support from the non-clinical PRx staff via (e.g., private staff-monitored Facebook group, group nutrition classes, one-on-one support from community health workers or health coaches). Automated text and email reminders are also utilized to encourage voucher redemption, class attendance, return for healthcare visits, and complete surveys. Seasonal recipes, tips, encouragement, and invitations to join the community advisory committee with options for video or telephone participation are also provided | Supplementary file 1 | N/A |
|  | **WHO PROVIDED** |  |  |
| **5.** | The Prx Vouchers were prescribed by any healthcare providers across the southern tier of New York, including nurses, primary care physicians, dietitians, and others. | Page 5 | <https://foodandhealthnetwork.org/prxproviders/>. |
|  | **HOW** |  |  |
| **6.** | In-person enrolment,  Vouchers were picked up at clinics, or vouchers were delivered if a telemedicine visit.  Text messaging for follow-up support.  Optional group education sessions. | Page 6, 7 | Supplementary file 1 |
|  | **WHERE** |  |  |
| **7.** | Conducted in multiple healthcare settings in Broome, Delaware, Tioga, and Tompkins counties | Page 6 | Supplementary file 1 |
|  | **WHEN and HOW MUCH** |  |  |
| **8.** | Duration: mid-June to November each year. Participants receive produce vouchers ($15/week), educational materials, and supportive messages. | N/A | Supplementary file 1 |
|  | **TAILORING** |  |  |
| **9.** | N/A | N/A | N/A |
|  | **MODIFICATIONS** |  |  |
| **10.^ǂ^** | N/A | N/A | N/A |
|  | **HOW WELL** |  |  |
| **11.** | Intervention adherence or fidelity was not formally assessed; however, all referring providers undergo an annual training/re-training, and have formal check-ins with the Program Manager at the start and the end of each season, and more frequently if needed. Food access is recorded via pick-up logs or delivery logs, which are tracked by the Program Manager. | N/A | N/A |
| **12.^ǂ^** | Intervention adherence or fidelity was not formally assessed. | N/A | N/A |

** **Authors** - use N/A if an item is not applicable for the intervention being described. **Reviewers** – use ‘?’ if information about the element is not reported/not sufficiently reported.

† If the information is not provided in the primary paper, give details of where this information is available. This may include locations such as a published protocol or other published papers (provide citation details) or a website (provide the URL).

ǂ If completing the TIDieR checklist for a protocol, these items are not relevant to the protocol and cannot be described until the study is complete.

* We strongly recommend using this checklist in conjunction with the TIDieR guide (see *BMJ* 2014;348:g1687) which contains an explanation and elaboration for each item.

* The focus of TIDieR is on reporting details of the intervention elements (and where relevant, comparison elements) of a study. Other elements and methodological features of studies are covered by other reporting statements and checklists and have not been duplicated as part of the TIDieR checklist. When a **randomised trial** is being reported, the TIDieR checklist should be used in conjunction with the CONSORT statement (see [www.consort-statement.org](http://www.consort-statement.org)) as an extension of **Item 5 of the CONSORT 2010 Statement.** When a **clinical trial** **protocol** is being reported, the TIDieR checklist should be used in conjunction with the SPIRIT statement as an extension of **Item 11 of the SPIRIT 2013 Statement** (see [www.spirit-statement.org](http://www.spirit-statement.org)). For alternate study designs, TIDieR can be used in conjunction with the appropriate checklist for that study design (see [www.equator-network.org](http://www.equator-network.org)).
